# The Jujube Genome Provides Insights into Genome Evolution and the Domestication of Sweetness/Acidity Taste in Fruit Trees

**DOI:** 10.1371/journal.pgen.1006433

**Published:** 2016-12-22

**Authors:** Jian Huang, Chunmei Zhang, Xing Zhao, Zhangjun Fei, KangKang Wan, Zhong Zhang, Xiaoming Pang, Xiao Yin, Yang Bai, Xiaoqing Sun, Lizhi Gao, Ruiqiang Li, Jinbo Zhang, Xingang Li

**Affiliations:** 1 College of Forestry, Northwest A&F University, Yangling, China; 2 Center for Jujube Engineering and Technology of State Forestry Administration, Northwest A&F University, Yangling, China; 3 Novogene Bioinformatics Institute, Beijing, China; 4 Boyce Thompson Institute, Cornell University, Ithaca, New York, United States of America; 5 College of Biological Sciences and Technology, Beijing Forestry University, Beijing, China; 6 Plant Germplasm and Genomics Center, Germplasm Bank of Wild Species in Southwest China, Kunming Institute of Botany, Chinese Academy of Sciences, Kunming, China; The University of North Carolina at Chapel Hill, UNITED STATES

## Abstract

Jujube (*Ziziphus jujuba* Mill.) belongs to the Rhamnaceae family and is a popular fruit tree species with immense economic and nutritional value. Here, we report a draft genome of the dry jujube cultivar ‘Junzao’ and the genome resequencing of 31 geographically diverse accessions of cultivated and wild jujubes (*Ziziphus jujuba* var. *spinosa*). Comparative analysis revealed that the genome of ‘Dongzao’, a fresh jujube, was ~86.5 Mb larger than that of the ‘Junzao’, partially due to the recent insertions of transposable elements in the ‘Dongzao’ genome. We constructed eight proto-chromosomes of the common ancestor of Rhamnaceae and Rosaceae, two sister families in the order Rosales, and elucidated the evolutionary processes that have shaped the genome structures of modern jujubes. Population structure analysis revealed the complex genetic background of jujubes resulting from extensive hybridizations between jujube and its wild relatives. Notably, several key genes that control fruit organic acid metabolism and sugar content were identified in the selective sweep regions. We also identified S-locus genes controlling gametophytic self-incompatibility and investigated haplotype patterns of the *S* locus in the jujube genomes, which would provide a guideline for parent selection for jujube crossbreeding. This study provides valuable genomic resources for jujube improvement, and offers insights into jujube genome evolution and its population structure and domestication.

## Author Summary

A balanced sweetness and acidity taste is among the most important characteristics of fruits. It is generally believed that human selection of sweetness plays a crucial role in the process of domestication from wild to cultivated fruit trees. However, the molecular mechanisms underlying fruit taste domestication still remain unclear. It is also unclear whether taste improvement is mainly determined by positive selection of advantageous traits such as sweetness or negative selection of disadvantageous trait such as acidity. Chinese jujube, domesticated from the wild jujube, is an economically important fruit tree crop in China. In this study, we sequenced and assembled the genome of a dry jujube and analyzed the genetic relationship between cultivated and wild jujubes through genome resequencing. Key genes involved in the acid and sugar metabolism were identified in the selective sweep regions. This finding suggested an important domestication pattern in fruit taste and also provided insights into the fruit molecular breeding and improvement.

## Introduction

Chinese jujube (*Ziziphus jujuba* Mill.) (2n = 2x = 24), native to China, is one of the oldest cultivated fruit trees, with more than 7,000 years of domestication history [1]. It belongs to the Rhamnaceae family in the Rosales order. Jujube is valued as a woody crop and traditional herbal medicine, and cultivated on 2 million hectares in China alone, with an annual production of approximately 4.32 million tons [2]. Jujube cultivars have been traditionally classified as fresh or dry, and dry jujubes account for approximately 80% of the total production. Ripe fruits of dry jujube have a coarse texture while those of fresh types have a crisp texture.

Cultivated jujubes were domesticated from their wild ancestors (*Z*. *jujuba* Mill. var. *spinosa* Hu.) through an artificial selection process for important agronomic traits, which resulted in architectural and structural changes in the tree such as a transition from bushes with more thorns to trees with fewer thorns and enlarged fruit sizes [1,3]. As with many agricultural crops, taste attributes of jujube fruits, such as sweetness and sourness, have been the subject of human selection. Fruits of cultivated jujubes have higher levels of sugars (up to 72% of the dry weight), while wild jujube fruits accumulate more soluble organic acids [3,4]. The domestication mechanism of fruit sweetness and acidity taste from their wild relatives is still not well characterized. Therefore, characterization of the sugar and acid metabolism of domesticated and wild jujubes through genome-wide analyses would help elucidate the genomic mechanism underlying fruit sweetness and acidity taste improvement.

The majority of jujube cultivars produce few seeds due to self-incompatibility or cross-incompatibility, which limit the practical artificial breeding of jujube. Gametophytic self-incompatibility (GSI) system is controlled by the *S* locus and has been found to operate in several *Ziziphus* species, including *Z*. *jujuba* [5–7]. Parents sharing the same S haplotype often result in seedless jujube kernels. Therefore, identification of the self-incompatibility locus (*S* locus) genes would provide a guideline to facilitate jujube breeding.

Recently, the draft genome of a fresh jujube cultivar ‘Dongzao’ with a high level of heterozygosity was reported, and it provides insights into the ascorbic acid metabolism and the adaptation mechanism to abiotic/biotic stresses [8]. However, little is known about jujube evolution, domestication, and the genetic bases of fruit quality. The genome sequencing of additional diverse jujubes would help us to address these questions, laying the foundation for improved strategies for jujube breeding. Here, we report the genome of a dry jujube cultivar ‘Junzao’ **Fig A and Fig B in S1 File,**). We also resequenced the genomes of 31 cultivated and wild jujube accessions with a range of geographical distributions. The genome sequences provided insights into the evolution of Rhamnaceae. Integrative transcriptome and resequencing analyses illuminated the genomic mechanisms underlying the domestication events of fruit sweetness and acidity.

## Results

### Genome sequencing, assembly and annotation

Sequencing of the ‘Junzao’ genome resulted in a 351-Mb assembly with contig and scaffold N50 sizes of 34 kb and 754 kb, respectively (**Table 1; Table A in S2 File**). A k-mer analysis of ‘Junzao’ sequences suggested an estimate genome size of ~350 Mb, consistent with the size estimated from the flow cytometry analysis (**Fig C in S1 File; Table B in S2 File**). The GC content of the assembled ‘Junzao’ genome was 32.6% (**Fig D in S1 File**). Approximately 98.3% of the 2,901 expressed sequence tag (EST) sequences and 98.9% of the assembled transcriptome contigs could be mapped to the ‘Junzao’ genome (**Table 1; Table C in S2 File**). In addition, 99.6% of the core eukaryotic genes were mapped to the ‘Junzao’ genome using CEGMA [9] (**Fig E in S1 File**) and 93.2% were completely mapped to the assembled ‘Junzao’ genome using BUSCO [10] (**Table 1**), indicating a high quality of the ‘Junzao’ genome assembly.

**Table 1:** Comparison of assembled genomes between ‘Junzao’ and ‘Dongzao’

	‘Junzao’ (LPXJ00000000)	‘Dongzao’ (JREP00000000) [8]
Tissues for DNA extraction	Leaves of mature tree (2n)	*In vitro* culture tissues
Estimate of genome size (Mb)	360	443
Chromosome number (2n)	2×12	2×12
Sequencing depth (×) ^a^	227	249
Total length of scaffolds (bp)	351,115,537	437,645,007
Anchored scaffolds (Mb)	293.7 (83.6%)	321.5 (73.6%)
N50 length (scaffolds ≥ 100 bp)	754,884	301,045
N50 length (contigs ≥ 100 bp)	34,020	33,948
ESTs covered by assembly (%)	98.3	94.9
CEGMA genes	247 (99.6%)	238 (96.0%)
BUSCO genes	891 (93.2%)	851 (89.0%)
Gene number	27,443	31,067 ^b^
Transposable elements (bp)	136,329,650 (38.8%)	204,918,483 (46.8%)
PAV-specific region (Mb)	14.2	7.8
PAV-specific genes	432	354

a. Calculated on the basis of the cleaned dataset.

b. Genes in the ‘Dongzao’ genome were predicted using the released scaffolds with the same method used for ‘Junzao’ gene predictions.

CEGMA, Core Eukaryotic Genes Mapping Approach; BUSCO, Benchmarking Universal Single-Copy Orthologs; PAV, presence-absence variation.

Using two high-density genetic linkage maps, we anchored 600 assembled scaffolds to the 12 linkage groups, covering 83.6% (293 Mb) of the assembled ‘Junzao’ genome (**Table D in S2 File; Fig F in S1 File**). We predicted a total of 27,443 protein-coding genes with an average coding sequence length of 1,136 bp and an average of 4.83 exons (**Table E in S2 File**), of which 91.2% were mapped to the 12 pseudo-chromosomes. A total of 2.1 million single-nucleotide polymorphisms (SNPs) were detected in the ‘Junzao’ genome, and therefore the heterozygosity level of the genome was calculated as 0.72% (**Table F in S2 File)**. In addition, 2,309 small insertions and deletions (indels) were found to be located in the exonic regions (**Table G in S2 File**).

### Comparison of the two jujube (‘Junzao’ vs. ‘Dongzao’) genomes

The assembled ‘Junzao’ genome was 86.5 Mb smaller than the reported genome of ‘Dongzao’ (437.7 Mb), which was assembled by sequencing the in vitro cultured plantlet [8]. One notable difference between the ‘Junzao’ genome and the reported ‘Dongzao’ genome was the abundance of transposable elements (TEs). A total of 136 Mb of TEs were identified, accounting for 38.8% of the assembled ‘Junzao’ genome, while the reported genome of ‘Dongzao’ contained 204 Mb of TEs (46.8%) (**Table 1; Fig G in S1 File; Table H and Table I in S2 File)**. In addition, a more recent accumulation of TEs was found in ‘Dongzao’ (<1.2 million years ago) (**Fig H(a) in S1 File**), and a greater proportion of genes were close to the TEs in ‘Dongzao’ than in ‘Junzao’ (**Fig H(b) in S1 File**). Phylogenetic analysis also indicated a greater expansion of specific LTR retrotransposon clades in the ‘Dongzao’ genome (**Fig H(c) in S1 File**).

Collinear genome regions between ‘Dongzao’ and ‘Junzao’ were identified (**Table J in S2 File**). The syntenic blocks in the ‘Dongzao’ genome (326.3 Mb) were 34.1 Mb larger than those in the ‘Junzao’ genome (292.2 Mb). We found that 26.0 Mb (77%) of the 34.1 Mb were repetitive sequences, further supporting that transposons are one of the major factors contributing to the genome size difference between ‘Junzao’ and ‘Dongzao’.

We found that unanchored scaffolds in the reported ‘Dongzao’ genome had many syntenic blocks with the anchored scaffolds, much higher than those in the assembled ‘Junzao’ genome (**Fig I in S1 File**). Furthermore, read coverage distribution of coding regions in the ‘Dongzao’ genome displayed a heterozygous peak at the half depth of the major homozygous peak, while no heterozygous peak was found in ‘Junzao’ (**Fig J. and Fig K in S1 File**). These findings suggest that sequences of heterozygous alleles from the same loci (redundant sequences) were sometimes failed to be assembled into consensus sequences in ‘Dongzao’, partially contributing to the larger genome assembly size of ‘Dongzao’ than that of ‘Junzao’. In addition, we identified ~4.9 Mb bacterial sequences in the ‘Dongzao’ genome assembly. Taken together, we suggest that higher levels of repetitive sequences, redundant sequences and bacterial contaminated sequences in the assembled ‘Dongzao’ genome have contributed to the larger genome assembly of ‘Dongzao’ than ‘Junzao’.

A presence-absence variation (PAV) analysis identified 7.8 Mb of ‘Dongzao’-specific sequences containing 354 genes and 14.2 Mb of ‘Junzao’-specific sequences containing 432 genes. Gene Ontology (GO) terms including DNA recombination and DNA integration were found to be significantly enriched in ‘Dongzao’-specific genes (**Table K in S2 File**). In addition, we identified 131 expanded gene families (930 genes) and 232 contracted families (702 genes) in ‘Junzao’ in comparison with ‘Dongzao’ (**Fig L in S1 File)**.

‘Junzao’ and ‘Dongzao’ are representative cultivars of dry and fresh jujubes, respectively (**Fig B in S1 File**). Their fruits contain highly different levels of crude fiber, which is derived from the fruit primary cell walls (**Table L in S2 File**). We found that several families of genes involved in cell wall modification were substantially expanded in the dry jujube ‘Junzao’ compared with ‘Dongzao’, including those encoding glycosyl hydrolases (beta-glucosidases, xyloglucan endotransglucosylase-hydrolases, endoglucanases and polygalacturonases) and those encoding pectin esterases and rhamnogalacturonate lyases (**Table M in S2 File**).

### Evolutionary scenario of genome rearrangements within the Rhamnaceae

**Fig 1:**
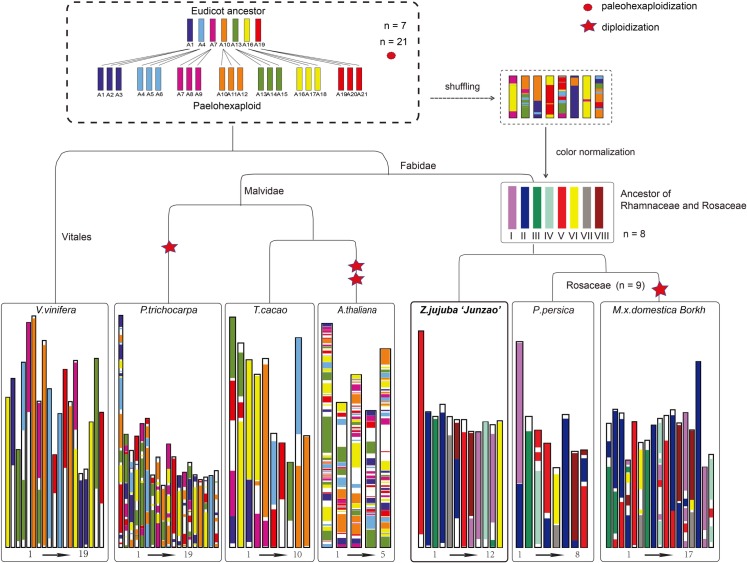
Evolutionary scenario of genome rearrangements from the ancestor of Rosales to jujube, peach and apple. In the top-right diagram, different colors in each chromosome represent the origin from the seven common ancestral chromosomes of eudicot, and the eight chromosomes filled with different colors represent putative paleo-chromosomes for the common ancestor of Rhamnaceae and Rosaceae.

Eight putative proto-chromosomes of the common ancestor of Rhamnaceae and Rosaceae, two sister families in the order Rosales, were inferred based on the available genome sequences of jujube, peach (*Prunus persica*) and apple (*Malus × domestica*) (**Fig 1**), and they are similar to the nine putative proto-chromosomes of the ancestor of Rosaceae [11,12]

No recent whole-genome duplication (WGD) events were detected in jujube [8] and peach [13] after their divergence, while one such event was identified in apple [14]. Although the numbers of proto-ancestral chromosomes in the Rosaceae increased from eight to nine after its divergence from the Rhamnaceae [11], we were still able to identify a one-to-two relation between the jujube and apple genomes. Considering the intergenomic relations among jujube, peach and apple, we determined the two largest chromosome synteny pairs as follows: 1) jujube chromosome 3, peach chromosome 2 and apple chromosomes 1 and 7, and 2) jujube chromosome 10, peach chromosome 3 and apple chromosomes 9 and 17 (**Fig 1**), which reflected the recent diploidization of the apple genome [14]. A conserved block was also identified among jujube chromosome 3, peach chromosome 2 and apple chromosome 7, which did not undergo any rearrangements, fissions or fusions and is thus likely derived directly from ancient chromosome III (**Fig 1**). These results showed that larger syntenic blocks were retained in jujube chromosomes, and illustrated that fewer chromosome fissions, fusions and rearrangements occurred in the jujube genome compared with the peach and apple genomes (**Table N in S2 File**).

### Jujube population structure

**Fig 2:**
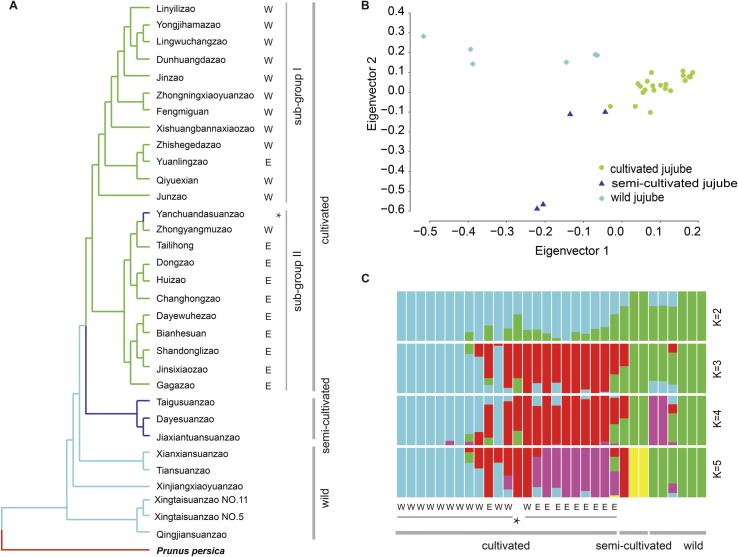
Jujube population structure. (A) Neighbor-joining phylogenetic tree of jujube accessions based on whole-genome SNP data. The jujube accessions are marked with E (east) or W (west), indicating their geographical distributions. (B) PCA of cultivated and wild jujube populations using whole-genome SNP data. (**C**) Population structure analysis of jujube accessions using FRAPPE at multiple kinship levels (K = 2, 3, 4 and 5). Each vertical bar represents one jujube accession. The length of each colored segment in each vertical bar represents the proportion contributed by ancestral populations. The accession marked with * in part A corresponds to the wild jujube ‘Yanchuandasuanzao’.

Resequencing the genomes of 31 accessions, including 10 wild jujube individuals (6 typical wild jujubes and 4 semi-wild accessions) and 21 jujube cultivars (**Table O in S2 File; Fig M in S1 File),** generated a total of 344 Gb of sequences, representing an average depth of 27.8× and an average coverage of 92.5% (**Table O in S2 File)**. After mapping the reads of each accession to the genome of ‘Junzao’, we detected a total of 5,300,355 SNPs. The parameter θπ values [15] indicated that wild jujubes, although represented in our analysis by half the number of accessions (10) as cultivated accessions (21), exhibited greater diversity (*θπ* = 2.60×10^−3^) than cultivated jujubes (*θπ* = 2.19×10^−3^). The neighbor-joining phylogenetic tree illustrated the domestication process as a transition from wild to cultivated jujubes via certain semi-wild accessions (**Fig 2A**). In addition, the cultivated jujube group could be further divided into two subgroups that were generally correlated with their geographical distributions in West China and East China (**Fig 2A; Fig N in S1 File**). A principle component analysis (PCA) generated a similar pattern (**Fig 2B**) to that of the phylogenetic analysis in that the jujube cultivars formed a tight cluster that was distant from the wild jujube accessions.

Population structure analysis indicated that wild and cultivated jujubes could be divided into two groups when K = 2, although admixed features were observed in 17 accessions covering both cultivated and wild jujubes (**Fig 2C**). With K = 3, the cultivated populations were further divided into two subgroups corresponding to their geographical distributions (West and East China), whereas the wild population remained relatively uniform. When the K value increased progressively from 3 to 5, new subgroups emerged in the wild jujube group and further differentiation was found within the cultivated jujubes (**Fig 2C**).

### Domestication of jujube and population differentiation

**Fig 3:**
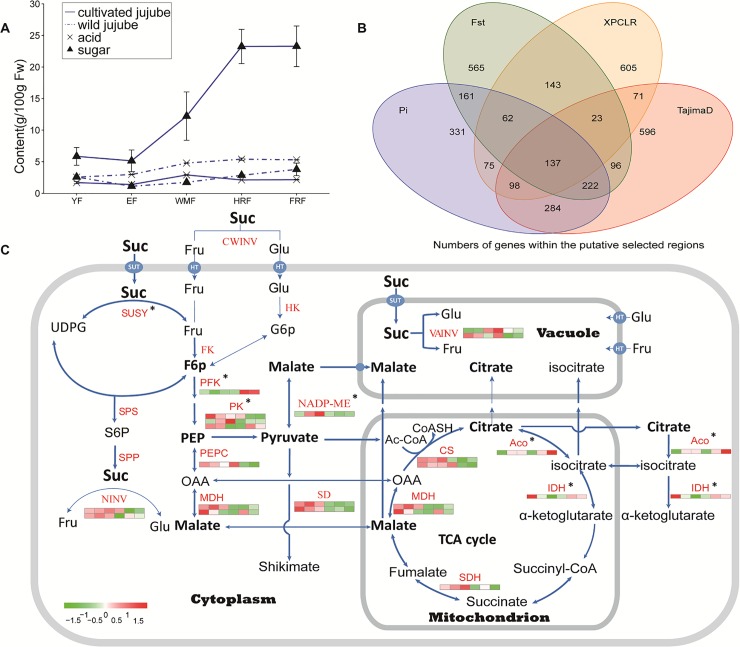
Jujube sugar and acid metabolism associated with domestication. **(A**) Sugar and acid accumulation in fruits at various developmental stages from cultivated and wild jujubes. YF, young fruit; EF, expanding fruit; WMF, white mature fruit; HRF, half-red fruit; and FRF, full-red fruit. (**B**) Number of genes detected in the putative selective regions using different methods. (**C**) Transcript abundance of genes involved in sugar and acid metabolism in cultivated and wild jujubes. Stars indicate the genes that were located in the regions putatively detected as selective sweeps. Scaled log_2_ expression values (RPKM) are shown in the heat map legend. The six boxes in one row of each heat map (left to right) correspond to the expression levels at stages EF, HRF and FRF of the wild accession (‘Qingjiansuanzao’) and WMF, HRF and FRF of the ‘Junzao’ cultivar. Each row in the heat map corresponds to one gene. SUSY: sucrose synthase; SPS: sucrose phosphate synthase; SPP: sucrose-phosphatase; HK: hexokinase; FK: fructokinase; PK: pyruvate kinase; MDH: malate dehydrogenase; ACO: aconitate hydratase; IDH: isocitrate dehydrogenase; SDH: succinate dehydrogenase; CS: citrate synthesis; NINV: neutral invertase; CWINV: cell wall invertase; vAINV: vacuolar acid invertase; PFK: 6-phosphofructokinase.

As shown in **Fig 3A**, jujube fruit had a much higher content of soluble sugars, and lower levels of organic acids than wild jujube fruits (**Table P in S2 File**), indicating both sweetness and acidity are important traits under human selection. Selective sweep regions covering 1,372 genes were identified in the jujube genome (**Fig 3B; Table Q in S2 File**). These included four genes, which encode an NADP-dependent malic enzyme (NADP-ME), a pyruvate kinase (PK), an isocitrate dehydrogenase (IDH), and an aconitate hydratase (ACO), all of which play key roles in organic acid metabolism in fruit (**Fig 3C; Table R in S2 File**). In addition, three vacuolar proton pumps (V-type proton ATPase), transporting H^+^ into vacuolar, were also in the putative sweep regions. On the other hand, three genes involved in sugar metabolism in fruit, encoding a sucrose synthase (SUSY), a phosphoglucomutase and a 6-phosphofructokinase, and 13 sugar transporters were also identified in the regions of putative selective sweeps.

Expression profiling analysis of sugar- and acid-related metabolism genes showed that a gene encoding a vacuole acid invertase (VAINV), an enzyme that irreversibly catalyzes the hydrolysis of sucrose to glucose and fructose, was expressed at a significantly lower level in the ripe fruits of cultivated jujubes than in those of wild jujubes, possibly contributing to higher sucrose accumulation in the vacuoles of cultivated jujube fruits (**Fig 3C**). In addition, most genes involved in acid metabolism pathways, including those encoding NADP-ME, PK, phosphoenolpyruvate carboxylase (PEPC), malate dehydrogenase (MDH), shikimate dehydrogenase (SD) and citrate synthesis (CS), were expressed at much higher levels in wild than in cultivated jujube fruits (**Table S in S2 File**). This trend was also the case for a neutral invertase in the sucrose biosynthesis pathway, which supplies glucose and fructose for organic acid metabolism (**Fig 3C**). On the contrary, genes involved in decomposing citrate, such as *ACO* and *IDH*, were expressed at lower levels in wild than in the cultivated jujubes.

Furthermore, a population differentiation analysis based on a population fixation index (*Fst*) between dry and fresh jujube groups (**Table T in S2 File**) uncovered four genes encoding beta-galactosidases and one encoding endo-1,4-beta-xylanase in the highly differentiated regions (**Table U in S2 File**).

### Identification of jujube *S*-locus genes

**Fig 4:**
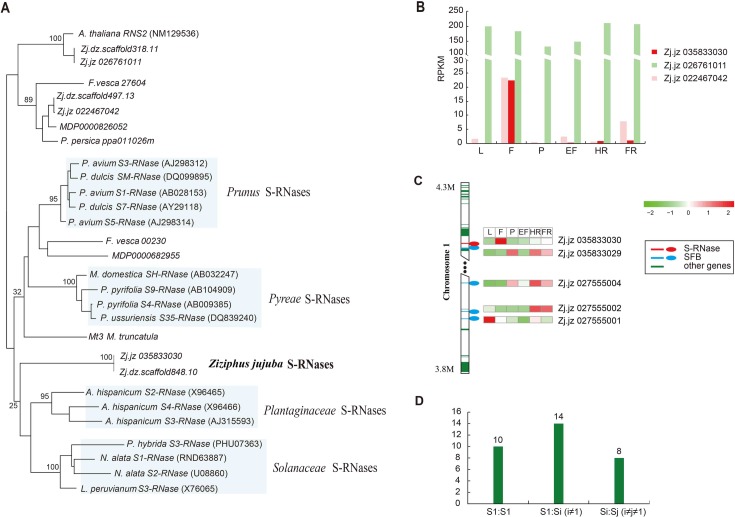
Characterization of the jujube *S* locus. **(A)** Phylogenetic analysis of three T2-RNase sequences from each ‘Junzao’ (Zj.jz) and ‘Dongzao’ (Zj.dz) with other known *S* genes. (**B)** Expression (RPKM) of *S*-RNase and *S*-like RNase genes in different tissues. **(C)** Physical positions of *S*-RNase and SFB genes on chromosome 1 of the ‘Junzao’ genome. The scaled log_2_ expression values (RPKM) are shown in the heat map. From left to right, leaf (L), flower (F), phloem (P), expanding fruit (EF), half-red fruit (HR) and full-red fruit (FR). (**D)** Frequency of *S*-locus genotypes in 32 jujube accessions.

We identified a candidate *S*-RNase gene (*Zj*.*jz035833030*; chromosome 1) and two *S*-like RNases (*Zj*.*jz026761011* and *Zj*.*jz022467042*; chromosomes 7 and 9, respectively) that belong to the T2-RNase family in the ‘Junzao’ genome (**Table V in S2 File**). We also identified the three T2-RNase genes in the ‘Dongzao’ genome (**Table V in S2 File**). Phylogenetic analysis further confirmed that *Zj*.*jz035833030* was the *S*-RNase and that *Zj*.*jz026761011* and *Zj*.*jz022467042* were *S*-like RNases (**Fig 4A)**. A transcriptome analysis revealed that *Zj*.*jz035833030* (named S1) was specifically expressed in flowers, and the two S-like RNase genes were expressed in all tested tissues (**Fig 4B**).

We identified four candidate SFB genes near the S1 S-RNase gene on chromosome 1 and inferred that the jujube *S* locus is likely localized within a narrow region (from 3.8–4.3 Mb) on chromosome 1. However, none of these four SFB genes was specifically expressed in flowers (**Fig 4C**). A phylogenetic analysis showed that all four SFB genes were clustered together in the same clade with the *Prunus* SFB (**Fig O in S1 File**). The structure of the jujube *S* locus was similar to that of pear [16].

By mapping genome sequencing reads that were generated from the 31 jujube accessions to the putative S1 region, we identified 21 SNPs (9 in the first exon, 11 in the second exon, and one in the intron) and 3 indels (one in the first exon, one in the second exon, and one in the intron). Among the 21 SNPs, 13 were located in the ribonuclease domain of T2-RNase (**Fig P in S1 File**). According to the SNP pattern in the *S*-RNase gene, we assigned each accession a corresponding haplotype. We found that S1 was the most common haplotype, and there were 10 accessions with a homozygous S1 locus and 14 with a heterozygous locus (**Fig 4D**).

## Discussion

We report the 351-Mb genome of the heterozygous dry jujube cultivar ‘Junzao,’ which is 86.5 Mb smaller than the assembled ‘Dongzao’ genome (437.7 Mb) [8]. We performed a series of analyses to characterize the size difference between the two genome assemblies, and revealed that the difference was primarily attributed to different levels of transposable elements, redundant sequences caused by heterozygosity, and bacterial contaminated sequences. In addition, we note that the k-mer and flow cytometry analyses of leaves from a ‘Dongzao’ mature tree indicated a similar genome size (~352 Mb) to that of ‘Junzao’ (**Table B and Table W in S2 File**). There are several reports of tissue culture-induced genome-level changes in plants, and one of the major underlying factors is the proliferation of transportable elements [17, 18]. Accordingly, the use of *in vitro* cultured plantlets as the source material for genome sequencing could lead to complex genetic background to some extent.

We resequenced a wide range of jujube cultivars and their wild relatives covering different fruit types (dry or fresh) and geographical distributions. This exploration revealed the various consequences of artificial selection during jujube domestication and elucidated the history of jujube domestication. Genome-wide SNP analysis revealed that naturally grown wild jujubes possessed higher diversity than cultivated jujubes, and the overall genetic diversity of jujube is similar to that of peach [19] but lower than that of several herbaceous species, such as soybean [20], rice [21] and cucumber [22]. A complex genetic background, caused by natural or artificial hybridization, was also revealed, which might explain why some semi-wild jujubes exhibited few traits that could distinguish them from cultivated jujubes, other than a sour taste and smaller fruit and tree sizes. Consistent with the population structure deduced by chloroplast diversity [23], our analyses using various approaches supported the hypothesis of an admixed population structure of wild and cultivated jujubes. We propose that this pattern reflects frequent exchanges of jujube germplasm between human populations from different sites and natural and artificial hybridizations.

The domestication of jujube has involved the selection for the fruit sourness and sweetness profiles, which are determined by acid and sugar metabolism. Analysis of fruit sugar and acid contents in a wide range of apple accessions suggested that fruit acidity rather than sweetness is likely to have undergone selection during apple domestication [24]. In this study, we found that unlike apple, sugar and acid metabolism were both undergone human selection during jujube domestication (**Fig 3A**). In particular, four genes and several sugar transporters involved in acid and sugar metabolism were determined to be putatively under human selection, of which two genes (*NADP-ME* and *PK*) are closely associated with the production of malate, which contributes directly to the acid taste of the fruit [25]. In addition, the expression levels of genes encoding enzymes, such as *NADP-ME*, *PK*, *PEPC* and *MDH* were substantially lower in cultivated jujube fruits in comparison with wild jujubes. Differential expression of the same gene sets between high-and low-acid apple fruits has also been reported [25, 26].

GSI have been characterized in the Rosaceae, Solanaceae and Plantaginaceae families which involves an *S* locus [27]. Although GSI is widely present in the *Ziziphus* species, the molecular mechanism controlling the hybridization behavior in jujubes is unknown. We identified the jujube *S* locus on the basis of genome sequence information, which was supported by gene expression analysis. Analyses of the S-locus genotypes of the 31 accessions provide guidelines for parent selection for crossbreeding. We have proved this strategy by crossing two random combinations with different *S* haplotypes, i.e., ‘Dongzao’ × ‘Linyilizao’ [28] and ‘Dongzao’ × ‘Zhongningyuanzao’ [29].

## Conclusions

We assembled a draft genome of the dry jujube cultivar ‘Junzao’ covering 351 Mb with contig and scaffold N50 sizes of 34 kb and 754 kb, respectively, which was 86.5 Mb smaller than that of ‘Dongzao’ (437.7 Mb) for which an *in vitro* culture plantlet was sequenced. Higher levels of repetitive sequences and redundant sequences in the assembled ‘Dongzao’ genome have primarily contributed to the larger genome assembly of ‘Dongzao’ than ‘Junzao’. By comparing the fresh (‘Dongzao’) and dry (‘Junzao’) jujube genomes, we found gene families involved in cell wall modification were largely expanded in ‘Junzao’, which might characterize the difference in fruit quality between dry and fresh cultivars. We reconstructed eight putative proto-chromosomes of the common ancestor of Rhamnaceae and Rosaceae based on the genome sequences of jujube, peach and apple, which elucidated the evolutionary processes that have shaped the genome structures of modern jujubes.

Genome resequencing of 31 geographically diverse accessions of cultivated and wild jujubes illustrated the domestication progress of jujubes and revealed the complex genetic background of jujubes caused by natural or artificial hybridizations. Based on the analysis of selective sweeps, we identified four genes involved in acidity metabolism pathways that encode an *NADP-ME*, *PK*, an *IDH*, and an *ACO*, all of which play key roles in organic acid metabolism. In addition, three V-type ATPase, enhancing organic acid storage in fruit, were also in the putative sweep regions. Furthermore, *SUSY* and several sugar transporter genes were determined to be putatively under selection. These findings might elucidate the changes in the sweetness/acidity taste caused by domestication events. We also identified the *S*-locus genes that controlled gametophytic self-incompatibility, and investigated the haplotype patterns of the *S* locus in diverse jujube accessions. Our study offers novel insights into the jujube population structure and domestication and provides valuable genomic resources for jujube improvement.

## Methods

### Plant materials, sequencing and assembly

A 9-year-old diploid, highly heterozygous dry jujube cultivar, ‘Junzao’ (voucher number: NWAFU-Junzao001), grown at the Jujube Experimental Station (N 37.13, E 110.09) of Northwest A&F University, Qingjian, Shaanxi Province, China, was used for genome sequencing. Genome sizes of jujubes including ‘Junzao’, ‘Dongzao’ and other 11 accessions were estimated by flow cytometry analyses on the young leaves (**Table B in S2 File**, **S1 File Supplementary notes**). The ‘Junzao’ genome was sequenced using a whole-genome shotgun strategy [30]. High-quality genomic DNA was extracted from young leaves using the Qiagen DNeasy Plant Mini Kit (Qiagen, Valencia, CA, USA). A total of 3 μg of DNA was used for each library construction. Short-insert paired-end libraries (180 bp and 500 bp) were generated using the NEB Next Ultra DNA Library Prep Kit for Illumina (NEB, USA) according to the manufacturer’s instructions. Large-insert (2 kb, 5 kb, 10 kb, 15 kb and 20 kb) DNA sequencing libraries were prepared through circularization by Cre-Lox recombination [31]. These libraries were sequenced on the Illumina HiSeq 2000 system. A total of 79 Gb of high-quality cleaned sequences (approximately 227x coverage of the genome) was generated and used for *de novo* genome assembly (**Table X in S2 File**). A modified version of SOAPdenovo was developed specifically for the *de novo* assembly of the highly heterozygous jujube genome (**S1 File Supplementary Notes**).

### Gene prediction

Augustus [32], Geneid [33], Genscan [34], GlimmerHMM [35] and SNAP [36] were used for *ab initio* gene predictions. We also aligned the protein sequences of *Arabidopsis thaliana*, *Capsicum annuum*, *Citrus clementina*, *Eucalyptus grandis*, *Malus* × *domestica*, *Oryza sativa*, *Populus trichocarpa*, and *Vitis vinifera* to the ‘Junzao’ genome using TBLASTN with an E-value cutoff of 1e^-5^. The homologous genome sequences were then aligned to the matched proteins for accurate spliced alignments using GeneWise [37]. Finally, a total of 36 Gb of high-quality RNA-Seq reads was aligned to the ‘Junzao’ genome using TopHat [38] with default parameters. Based on the RNA-Seq read alignments, Cufflinks [39] was then used for transcriptome-based gene structure predictions. Outputs from *ab initio* gene predictions, homologous protein alignments and transcript mapping were integrated using EVM [40] to form a comprehensive and non-redundant reference gene set and then filtered by removing the genes with incorrect coding sequences and putative repeat elements (80% coverage).

### Genetic map construction and scaffold anchoring

We combined two genetic maps, described below, to anchor the assembled scaffolds of ‘Junzao.’ First, we used a previously published restriction site-associated DNA (RAD)-based high-density genetic map generated from an inter-specific F_1_ population to anchor the genome assembly [41]. Second, we constructed a genetic map by using a different F_1_ population (‘Dongzao’ × ‘Yingshanhong’, 96 progenies), which was also based on the RAD strategy according to Baird et al [42]. High-quality SNP and SSR markers were used to construct a linkage map (**Table D in S2 File**). The resulting genetic map was used to further anchor the assembled scaffolds of ‘Junzao.’

### Genome evolution analysis

To better understand the evolutionary processes that shaped the genome structures of jujubes, we reconstructed the putative proto-chromosomes of the common ancestor of Rhamnaceae and Rosaceae, which are sister families in the order Rosales [43]. Protein sequences from 13 plant species (*A*. *thaliana*, *C*. *annuum*, *C*. *sinensis*, *M*. × *domestica*, *O*. *sativa*, *P*. *trichocarpa*, *V*. *vinifera*, *Cucumis sativus*, *Pyrus × bretschneideri*, *Actinidia chinensis*, *Cypripedium arietinum*, *Z*. *jujuba* ‘Dongzao’ and *Z*. *jujuba* ‘Junzao’) were extracted for building gene families. For alternatively spliced isoforms, only the longest proteins were used in the analysis. An all-to-all BLASTP was used to compare protein sequences with an E-value cutoff of 1e^-7^, and OrthoMCL [44] was then used to cluster genes from these species into families with the parameter “-inflation 1.5.” MUSCLE [45] was used to generate multiple sequence alignments of proteins in single-copy gene families with default parameters. RAxML [46,47] and a ‘supermatrix’ of protein sequences were used to construct the phylogenetic tree with the maximum likelihood algorithm. A molecular clock model was implemented to estimate the divergence time of these 13 species using McMctree in PAML [48]. To obtain a more accurate result, ‘r8s’ was used to estimate the divergence time based on the constructed tree. Café [49] was used to identify gene families that have undergone significant expansion or contraction in the *Z*. *jujuba* ‘Junzao’ genome with a p-value cutoff of 0.05.

### Collinear region and PAV detection between ‘Junzao’ and ‘Dongzao’ genomes

The one-to-one collinear regions between ‘Junzao’ (accession number: PRJNA306374) and ‘Dongzao’ [8] were detected using the MUMmer package [50] with the parameters ‘-maxmatch -c 90 -l 40 -d 0.05’. The sequence alignments were performed on the scaffold level between these two jujube genomes. The best reciprocal alignments with length less than 300 bp or an identity less than 90% were discarded, and then the aligned regions within the same scaffold were connected together. Regions were identified as syntenic blocks if there were more than 5 adjacent alignment regions between the two genome sequences.

In addition, we use the “show-snps” program in MUMmer package to detect homozygous SNPs and small indels from the one-to-one alignments. RepeatMasker (http://www.repeatmasker.org/RepeatModeler.html) was used to find repeat elements in sequences that could not be aligned to the ‘Junzao’ genome. Sequences shorter than 100 bp were removed. ‘Dongzao’-specific sequences were obtained after realigning them with the ‘Junzao’ genome and discarding sequences with identities of greater than 95% and gap lengths of less than 100 bp. Potential bacterial sequences, which were identified on the basis of BLAST searches against the GenBank NT database, were excluded. Genes with at least 90% of the CDS regions covered by ‘Dongzao’-specific sequences were defined as ‘Dongzao’-specific genes.

### Reconstruction of common ancestral chromosomes

Syntenic blocks shared by the seven species (*V*. *vinifera*, *P*. *trichocarpa*, *Theobroma cacao*, *A*. *thaliana*, *Prunus persica*, *M*. *× domestica*, and *Z*. *jujuba* ‘Junzao’) were identified with MCscanX [51] using the grapevine genome as the reference. Syntenic blocks containing at least 3 gene pairs were retained to reconstruct the genome structure of the seven selected species. Based on the syntenic and overlapping relations of *Z*. *jujuba*, *P*. *persica* and *M*. *× domestica* genomes, we reconstructed the paleo-chromosomes of the common ancestor of Rhamnaceae and Rosaceae using a previously described method [52, 53]. The structures of the *V*. *vinifera*, *P*. *trichocarpa*, *T*. *cacao* and *A*. *thaliana* genomes were reconstructed by comparing them with the seven proto-chromosomes of the eudicot ancestor.

### Resequencing and SNP calling

Thirty-one jujube accessions were chosen for genome resequencing analysis, including 10 wild jujube individuals (6 typical wild jujubes and 4 semi-wild accessions) and 21 jujube cultivars (**Table O in S2 File; Fig M in S1 File)**. Illumina paired-end genome libraries were constructed for each accession following the manufacturer’s instructions and then sequenced on Illumina HiSeq 2500/4000 platforms, which yielded a total of 363 Gb of raw paired-end sequences. The raw data were processed to remove low-quality bases, adapter sequences, and putative PCR duplicates, resulting in a total of 344 Gb of high-quality paired-end sequences. The cleaned reads were mapped to the ‘Junzao’ genome using BWA [54] with the parameters “mem -t 4 -k -M” [29]. BAM files were processed for SNP calling using the SAMtools mpileup function [55] with the parameters “-m 2 -F 0.002 -d 1000 -u.” High-quality SNPs, which were supported by a coverage depth of 5–1,000, mapping quality >20, distance of adjacent SNPs >10 bp and missing ratio of samples within each group <50%, were retained for subsequent analyses.

### Phylogenetic analysis and population structure of jujube accessions

Peach was used as the outgroup to construct the phylogenetic tree. The NUCmer program in MUMmer [50] was used to align the peach genome (GenBank accession no. AKXU00000000) with the ‘Junzao’ genome with default settings. SNPs within the best-hit regions were extracted, and then the genotypes of peach were used to provide outgroup information at corresponding positions. The neighbor-joining phylogenetic tree was constructed using Treebest-1.9.2 (http://treesoft.sourceforge.net/treebest.shtml) on the basis of the p-distance. We used the parameter θπ [15] to assess the level of genetic diversity for cultivated and wild jujube populations by scanning whole-genome SNP sites, respectively. PCA was performed using EIGENSOFT [56]. The eigenvectors were obtained from the covariance matrix using the R function ‘eigen.’ The population structure was further inferred using the program FRAPPE [57] with kinship (K) set from 2 to 5 and the maximum iteration of expectation-maximization set to 10,000.

### Identification of selective sweeps and population differentiation

Four methods, i.e., the θπ ratios, pairwise population differentiation (*Fst*) levels [58], Tajima’s D test [59] and the cross-population composite likelihood ratio test (XP-CLR) [60], were used to identify the selective sweeps associated with jujube domestication events. Briefly, θπ ratios (θπ, wild/θπ, cultivated), *Fst* and TajimaD were calculated using a sliding window analysis with a window size of 20 kb and a step size of 10 kb. XP-CLR test was performed with the following parameters: sliding window size of 0.6 cM, grid size of 10 kb, maximum number of SNPs within a window of 300, and correlation value for 2 SNPs weighted with a cutoff of 0.95. Genome regions with the top 5% of scores in each of the four methods were identified and those detected by at least two of the methods were identified as selective sweeps. In addition, we used the top 5% highest *F*_*ST*_ values to characterize the population differentiation between dry and fresh jujubes. Genes within these regions were subjected to GO enrichment analysis using EnrichPipline [61].

### *S* locus gene identification and analysis

We compared the ‘Junzao’ genes to the information in a local database containing known *S*-RNase gene sequences collected from NCBI with an E-value cutoff of 1e^-10^ using BLASTN. We then screened for genes belonging to the T2 RNase gene family from the BLAST results because *S*-RNase genes are members of the T2 RNase family [62, 63]. Candidate *S*-RNase genes were further screened according to two criteria: the absence of the amino acid pattern 4 ([CG] P [QLRSTIK][DGIKNPSTVY]) [63] and the presence of a maximum of two introns [64]. The *S*-RNase genes from four families (Rosaceae, Fabaceae, Solanaceae, and Plantaginaceae) and the candidate jujube *S*-RNases were used to construct the phylogenetic tree using RAxML with a generalized time-reversible (GTR) model of sequence evolution.

Pollen-determinant *S*-haplotype-specific genes belong to the F-box family. We used a similar BLAST strategy to that described above to search for the F-box genes in the chromosome region in which the candidate *S*-RNase gene was located. A phylogenetic analysis was performed for those candidate SFB genes together with the known *Prunus* SFB, *Petunia* SLFs, *Prunus* SLF1 and *Malus* SFBs.

To investigate the expression of the candidate *S*-RNase and SFB genes, we used RNA-Seq data from leaves, phloem, flowers and fruits of ‘Junzao.’ The SNP calling results derived from the resequencing of 31 accessions were used to reconstruct the two haplotypes of *S*-RNase gene using HapCUT [65].

### Transcriptome sequencing and expression analysis

Phloem, mature leaves, flowers, and fruits at different stages (expanding fruit, half-red, and full-red) of the ‘Junzao’ cultivar and the wild jujube ‘Qingjiansuanzao’ (8 years old) were collected in 2013 and 2014, respectively. All the samples were immediately frozen in liquid nitrogen. Total RNAs were isolated using a modified CTAB method and then treated with RNase-free DNase I (Promega, USA). First-strand cDNAs were synthesized using a Clontech kit. RNA-Seq libraries were constructed using the NEB Next UltraTM RNA Library Prep Kit (NEB, USA) and sequenced on a HiSeq 2000/2500 system. RNA-Seq reads were mapped to the ‘Junzao’ genome using TopHat [38]. The total numbers of aligned reads (read counts) for each gene were normalized to the reads per kilobase exon model per million mapped reads (RPKM) [66]. DESeq [67] was used to identify differentially expressed genes.

### Determination of sugar and organic acid levels in jujube fruits

Fruits were collected at three developmental stages: expanding fruit, half-red fruit and full-red fruit. Sugars (fructose, glucose and sucrose) and acids (malic acid, citric acid and succinic acid) were quantified using high-performance liquid chromatography (HPLC, Shimadzu) as described previously [68]. A total of 1 g of the edible part of the dried jujubes was ground and incubated in 50 mL of 80% ethanol in an ultrasonic bath (40 kHz, 45°C, 20 min). The samples were centrifuged at 3,500 ×*g* for 10 min, and the supernatant was collected in a new tube. The pellet was re-extracted by repeating the above steps. The combined supernatants were evaporated in a rotary evaporator at 45°C and then diluted with deionized water to 10 mL. The diluted extracts were filtered through a 0.45-μm membrane filter prior to HPLC analysis.

### Data availability

Accession codes: Sequence data have been deposited in the GenBank/EMBL/DDBJ nucleotide core database under the accession number LPXJ00000000 (PRJNA306374) and all sequence reads have also been deposited in the online database. The version described in this paper is the first version.

## Supporting Information

S1 File Supplementary Notes and Figures. **Fig A. Cultivation and morphological characteristics of the dry cultivar ‘Junzao’.** (a) Cultivation of ‘Junzao’ in arid desert conditions with a wide row planting pattern (3.5m × 1m). (b) A ‘Junzao’ tree of more than 600 years old, in Jiaocheng, Shanxi Province. (c) Developing fruits at the expanding stage; (d) Fruits at the full red mature stage. (e) Naturally dried fruits after fully maturing on the tree. (f) Major development stages of jujube fruit and dried fruit after fully maturing. **Fig B. Fruits during ripening and post-harvest storage of a typical dry cultivar (‘Junzao’) and a fresh cultivar (‘Dongzao’).** During the softening stage, fruits of the dry-cultivar ‘Junzao’ shrink while fruits of the fresh cultivar ‘Dongzao’ decay and do not reach the dried stage. **Fig C. Frequency distribution of all 19-mers and heterozygous 19-mers of ‘Junzao’. Fig D. GC content and sequencing depth.** (a) A major island in the scatter graph of the distribution of GC content against sequencing depth indicated no contamination from other species in ‘Junzao’, (b) A small cluster (indicated with a circle) distant from the major island presented in ‘Dongzao’. **Fig E. Mapping results of four core eukaryotic gene subsets to the genomes ‘Junzao’, ‘Dongzao’, *M*. × *domestica* and *P*. *trichocarpa***. The total set of core eukaryotic genes was divided into four groups according to their degree of protein sequence conservation. **Fig F. Anchoring the ‘Junzao’ assembled scaffolds to genetic maps.** The ‘Junzao’ assembled scaffolds were anchored to the 12 linkage groups (LG1-LG12, red) using two high-density genetic linkage maps. A total of 208 Mb (green, 59.28% of the assembled genome) were anchored by both maps, 71 Mb (Blue, 20.48%) were anchored only by the genetic map reported by Zhao et al., and 13 Mb (yellow, 3.86%) were anchored only by the genetic map constructed in this study. **Fig G. Divergence rate of transposable elements in the *Z*. *jujuba* ‘Junzao’ genome. Fig H. Comparison of transposable elements between the two genome sequences of *Ziziphus jujuba*.** (a) Insertion time of long terminal repeat (LTR) retrotransposons in the ‘Dongzao’ and ‘Junzao’ genomes. The insertion time was estimated using the formula: T (time) = K/(2*r), where K represents the average number of substitutions per aligned site and r represents the average substitution rate, which was assigned as 1.3e-8 substitutions per synonymous site per year. (b) Distance from individual TEs to their closest genes. (c) Phylogeny of Ty1/copia-like and Ty3/gypsy-like LTR retrotransposons. **Fig I. Orthologous gene blocks between anchored scaffolds and unanchored scaffolds in ‘Dongzao’ (left) and ‘Junzao’(right) Fig J. Average sequencing depth distribution of ‘Dongzao’ genes**. All the cleaned reads generated by sequencing genomic DNA derived from a mature ‘Dongzao’ tree were mapped to the assembled ‘Dongzao’ genome and the average sequencing depth of genes was plotted (orange). For most of genes, the sequencing depth was 36×. However, we observed a secondary peak in the distribution at half of the average sequencing depth (18×). The average sequencing depth of the 2,615 genes from 1,126 families containing fewer gene members in ‘Junzao’ than ‘Dongzao’ was ~18× (blue). **Fig K. Read coverage distribution of coding regions in ‘Junzao’ and the previous reported ‘Dongzao’.** ‘Dongzao’ reads were generated by sequencing genomic DNA derived from a mature ‘Dongzao’ tree and mapped to the previous reported ‘Dongzao’ gene models as described in Fig J. **Fig L. Phylogenetic tree and gene family expansion and contraction.** The phylogenetic tree was constructed from a concatenated alignment of 205 single-copy gene families from 12 eudicots and *O*. *sativa*. Gene family expansions are indicated in green, and contractions are indicated in red; the corresponding proportions among total changes are shown using the same colors in the pie charts. **Fig M. Fruits of the jujube accessions used the in phylogenetic analysis. Fig N. Geographical location of the jujube cultivars and wild jujube accessions used in the resequencing analyses.** Cultivated jujubes sampled from East China are marked by red circles (E), those from West China by green circles (W), and the wild accessions by blue circles (Other). **Fig O in S1 Fil.e Phylogenetic tree of predicted SFB genes in Z. jujuba and SFB, SLF and SLF-like genes identified in *Malus* x *domestica*, *Prunus persica*, *Prunus mume*, *Fragaria vesca*, and *Fragaria nipponica*.** The tree was rooted with *A*. *thaliana* F-box/kelch-repeat gene (NM111499). Phylogenetic tree was constructed using RAxML with the Generalised Time-Reversible (GTR) model of sequence evolution. **Fig P. SNPs and indels identified in the candidate S-RNase gene (Zj.jz035833030) based on resequencing results.** Bases with green background indicate the ribonuclease domain of T2-RNase.
(DOCX)

S2 File Supplementary Tables. Table A. Summary of ‘Junzao’ genome assembly. Table B. Genome size estimation using flow cytometry. Table C. Assessment of *Z*. *jujuba* ‘Junzao’ genome assembly using EST sequences and assembled transcriptome contigs. Table D. Summary of ‘Junzao’ scaffold anchoring to the linkage maps. Table E. Statistics of predicted protein-coding genes in *Z*. *jujuba* ‘Junzao’. Table F. Summary of identified heterozygous SNPs in the genome of *Z*. *jujuba* ‘Junzao’. Table G. Summary of small indels resulting in stop codon gain/loss or frameshift. Table H. Summary of identified repeats in the genome of *Z*. *jujuba* ‘Junzao’. Table I. Classification of transposable elements in the genome of *Z*. *jujuba* ‘Junzao’. Table J. Comparisons of syntenic regions and repeat contents between the genomes of ‘Dongzao’ and ‘Junzao’. Table K. GO terms significantly enriched in genes associated with ‘Dongzao’ PAVs. Table L. Comparison of jujube fruit quality between dry and fresh cultivars. Table M. Number of genes involved in the cellulose degradation in the expanded families found in ‘Junzao’ compared with ‘Dongzao’. Table N. Average number of break points per chromosome in the three species. Table O. Cultivated and wild jujube accessions selected for re-sequencing analysis. Table P. Sugar and acid content at different stages of jujube fruit development (g/100 g FW). Table Q. Genes within the putative selected regions identified by four methods. Table R. Genes in selected regions related to sugar/acid metabolism and accumulation. Table S. Expression profiles of genes involved in sugar/acid metabolism in fruits of wild jujube (‘Qingjiansuanzao’) and cultivated jujube (‘Junzao’). Table T. Dry and fresh jujubes selected for the *Fst* analysis. Table U Genes in the highly differentiated regions that were related to the coarse and crisp textures of dry and fresh jujubes. Table V Information of the T2-RNase gene predicted in ‘Junzao’ and ‘Dongzao’. Table W Genome size estimation of *Ziziphus jujuba* ‘Dongzao’ by sequencing the mature tree at 34× depth. Table X Summary of ‘Junzao’ genome sequencing data.
(XLSX)
