# Supplementary material for: The Jujube Genome Provides Insights into Genome Evolution and the Domestication of Sweetness/Acidity Taste in Fruit Trees
Source: PLoS Genet. 2016 Dec 22;12(12):e1006433. doi: 10.1371/journal.pgen.1006433 (PMC5179053; doi:10.1371/journal.pgen.1006433)
Supplement: S1 File — Fig A. Cultivation and morphological characteristics of the dry cultivar ‘Junzao’. (a) Cultivation of ‘Junzao’ in arid desert conditions with a wide row planting pattern (3.5m × 1m). (b) A ‘Junzao’ tree of more than 600 years old, in Jiaocheng, Shanxi Province. (c) Developing fruits at the expanding stage; (d) Fruits at the full red mature stage. (e) Naturally dried fruits after fully maturing on the tree. (f) Major development stages of jujube fruit and dried fruit after fully maturing. Fig B. Fruits during ripening and post-harvest storage of a typical dry cultivar (‘Junzao’) and a fresh cultivar (‘Dongzao’). During the softening stage, fruits of the dry-cultivar ‘Junzao’ shrink while fruits of the fresh cultivar ‘Dongzao’ decay and do not reach the dried stage. Fig C. Frequency distribution of all 19-mers and heterozygous 19-mers of ‘Junzao’. Fig D. GC content and sequencing depth. (a) A major island in the scatter graph of the distribution of GC content against sequencing depth indicated no contamination from other species in ‘Junzao’, (b) A small cluster (indicated with a circle) distant from the major island presented in ‘Dongzao’. Fig E. Mapping results of four core eukaryotic gene subsets to the genomes ‘Junzao’, ‘Dongzao’, M. × domestica and P. trichocarpa. The total set of core eukaryotic genes was divided into four groups according to their degree of protein sequence conservation. Fig F. Anchoring the ‘Junzao’ assembled scaffolds to genetic maps. The ‘Junzao’ assembled scaffolds were anchored to the 12 linkage groups (LG1-LG12, red) using two high-density genetic linkage maps. A total of 208 Mb (green, 59.28% of the assembled genome) were anchored by both maps, 71 Mb (Blue, 20.48%) were anchored only by the genetic map reported by Zhao et al., and 13 Mb (yellow, 3.86%) were anchored only by the genetic map constructed in this study. Fig G. Divergence rate of transposable elements in the Z. jujuba ‘Junzao’ genome. Fig H. Comparison of transposable elements [file pgen.1006433.s001.docx]

# The jujube genome provides insights into genome evolution and the domestication of sweetness/acidity taste in fruit trees

Jian Huang^1,2^*, Chunmei Zhang^1,2^*, Xing Zhao^3^*, Zhangjun Fei^4^*, KangKang Wan^3^, Zhong Zhang^1,2^, Xiaoming Pang^5^, Xiao Yin^1^, Yang Bai^4^, Xiaoqing Sun^3^, Lizhi Gao^6^, Ruiqiang Li^3^, Jinbo Zhang^3^, Xingang Li^1,2^

Jian Huang (huangj@nwsuaf.edu.cn)

Chunmei Zhang (mei_lily@nwsuaf.edu.cn)

Zhong Zhang (zhangzhong@nwsuaf.edu.cn)

Xiao Yin (insmile@nwsuaf.edu.cn)

Xingang Li (xingangle@nwsuaf.edu.cn)

^1^College of Forestry, Northwest A&F University, Yangling, 712100, China

^2^Center for Jujube Engineering and Technology of State Forestry Administration,

Northwest A&F University, Yangling, 712100, China

Xing Zhao ([zhaoxing@novogene.com](mailto:zhaoxing@novogene.com))

Kangkang Wan ([wankangkang@novogene.com](mailto:wankangkang@novogene.com))

Xiaoqing Sun (sunxiaoqing@novogene.com)
Ruiqiang Li (liruiqiang@novogene.com)

Jinbo Zhang (zhangjinbo@novogene.com)

^3^Novogene Bioinformatics Institute, Beijing 100083, China

Zhangjun Fei (zf25@cornell.edu)

Yang Bai (yb63@cornell.edu)

^4^Boyce Thompson Institute, Cornell University, Ithaca, New York 14853, USA.

Xiaoming Pang (xmpang@163.com)

^5^College of Biological Sciences and Technology, Beijing Forestry University, Beijing 100083, China

Lizhi Gao (lgao@mail.kib.ac.cn)

^6^ Plant Germplasm and Genomics Center, Germplasm Bank of Wild Species in Southwest China, Kunming Institute of Botany, Chinese Academy of Sciences, Kunming 650201, China

*These authors contributed equally to this work.

Correspondence should be addressed to Xingang Li (xingangle@nwsuaf.edu.cn), Jinbo Zhang (zhangjinbo@novogene.com)

**Supplementary notes** 3

1. Description of *Ziziphus jujuba* ‘Junzao’ 3

2. Difference in fruit quality between dry and fresh cultivars 3

3. Estimation of jujube nuclear genome size by flow cytometric analyses 4

3.1 Sample preparation 4

3.2 Preparation of nuclei suspensions 4

3.3 Flow cytometric analyses 4

3.4 Genome size estimation 5

4. Filtering of Illumina sequencing reads 5

5. Genome assembly 5

6. Quality assessment of genome assemblies 7

7. Repeat annotation 7

8. Gene annotation 8

**Supplementary Figures** 9

References 25

# Supplementary notes

## 1. Description of *Ziziphus jujuba* ‘Junzao’

‘Junzao’ is the most widely cultivated dry jujube cultivar in China, with an annual production of ~2 million tons, accounting for 45% of the total jujube yield, as determined by China Forestry Bureau (**Fig A**). With a harvest area of 0.37 million hectares (~35% of the total cultivated area), ‘Junzao’ had an annual output value of 20 billion Yuan (RMB) in 2013. ‘Junzao’, a landrace of Shanxi Province, has been cultivated for over 2,000 years, and many trees older than 800 years can still be found in Jiaocheng County (37.57 N 112.13 E) and Taigu County (37.39 N 112.55 E), Shanxi Province. Over the last decade, ‘Junzao’ has been introduced into the Xinjiang Autonomous Region, where it has become the dominant cultivar, with over 0.33 million hectares, which can be attributed to its significantly improved fruit quality than that in its origins [1].

During the fruit growth period (ca. 110 days), five main developmental stages have been identified, i.e., young, expanding, white mature, crisp mature and softening stages ( **Fig A(d)**). In the crisp mature stage, ‘Junzao’ fruit has a long obovate shape, a dark red color and an average mass of ~26.3 g (up to 50.0 g), an edible rate of 96.3%, a soluble solid content of 33%, a total sugar content of 28.7%, a total acid content of 0.45% and a vitamin C content of 430.2 mg/100g. Mature fruits naturally dry on the tree in arid dessert environments, but rarely remain on the tree in the reaches of the Yellow River of China, a traditional jujube production area. After drying, the fruit became plump, with a high sugar (71.8% of dry weight) and low acid (1.58% of dry weight) content [2].

## 2. Difference in fruit quality between dry and fresh cultivars

Jujube cultivars were traditionally divided into two types, fresh and dry, according to fruit usage. Significant differences in fruit quality between fresh and dry cultivars can be easily observed (**Table L in S2 File**) [3]. Due to their crisp texture, and sweeter taste, fresh cultivars show better fruit commodity performance at the crisp ripening stage (from beginning red to full red) than the dry cultivars. However, their fruit can decay rapidly after reaching the full red stage or during postharvest (**Fig B**). In contrast, fruits of dry cultivars are propitious to be made into dried fruits after full maturity, which might be attributable to the higher content of crude fiber and lower moisture levels (**Table L in S2 File**). During the softening and drying process, components of fruit cell wall degrade and the fruit accumulates sugars and polysaccharide that are noted for its medicinal properties [4,5].

## 3. Estimation of jujube nuclear genome size by flow cytometric analyses

### 3.1 Sample preparation

A total of 4 wild jujubes and 9 jujube cultivars were selected for flow cytometry analyses (**Table B in S2 File)**, all of which were planted in the jujube experimental station of Northwest A&F University in Qingjian County, Shaanxi Province, China. Seedlings of Nipponbare (*Oryza sativa* L.) were used as the internal reference.

### 3.2 Preparation of nuclei suspensions

Suspensions of intact nuclei were prepared according to Huang et al. [6]. Briefly, ~50 mg young leaves were harvested from the young shoots, shredded into small pieces and immersed in 1 ml cold isolation buffer. The homogenate was filtered through a 50 µm nylon filter and then subjected to centrifugation. The precipitate was re-suspended using the Otto buffers [100mM citric acid; 0.5 % (v/v) Tween 20 (pH ~2.3), and then 10 μl 50 μg·ml^-1^ RNases and 80 μl 1 mg·ml^-1^ propidium iodide (PI) (Sigma, St. Louis, MO, USA) were added for nuclear staining.

### 3.3 Flow cytometric analyses

The fluorescence detection was performed on a FACSCalibur^TM^ Flow Cytometer (BD, USA). The instrument was equipped with an air-cooled argon-ion laser (15 mW, 488 nm). PI fluorescence was collected through a 645-nm dichroic long-pass filter and a 620-nm band-pass filter. The amplifier system was set to a constant voltage and 20,000 nuclei were analyzed for each sample. The results of the flow cytometry were analyzed using the Cellquest software and gated to selectively visualize all cells of interest that gather densely in a dotplot map, while eliminating results from unwanted particles. The average of coefficient of variation values (CV) was used to evaluate the results, and those with a CV< 5% were considered to be reliable. Here, CV = D/M×l00%, where D is the standard deviation of the cell distribution and M is the average of cell distribution. Nuclear DNA content was calculated as a linear relationship between the ratio of 2C-value peaks of the sample and the standard. For each sample, four replicates were performed.

### 3.4 Genome size estimation

The 2c nuclear DNA content was calculated with the following formula:

$$2C nuclear DNA content=$$

$$\frac{\mathrm{sample}{G0}/{G1} mean FL}{reference standard {G0}/{G1 mean FL}}\times2C nuclear DNA content of reference standard$$

## 4. Filtering of Illumina sequencing reads

After removing the adapter sequences, low quality and duplicated reads, the following types of reads were also removed:

(1) Reads having an ‘N’ >10% of its length.

(2) Reads containing > 10 bp of the adapter sequence.

(3) Read pairs with identical left and right reads (thus considered to be the products of PCR duplication).

(4) Reads with a k-mer frequency <4 after correction (to minimize the influence of sequencing errors).

## 5. Genome assembly

First, all 19-mers in the paired-end reads were counted to generate a 19-mer frequency distribution (**Fig C**). Two peaks were observed: one heterozygous peak at 57x and one homozygous peak at 114x. According to the 19-mer frequency, we chose 19-mers with depths ranging from 10 to 90 as the initial heterozygous 19-mer set. All the paired-end reads were also split into 37-mers to build the *de Bruijn* graphs and perform the contig assembly using SOAPdenovo [7] with the parameters ‘-d 1 -R -M 0’. We mapped the 19-mers to the contigs and removed non-heterozygous 19-mers from the above initial heterozygous 19-mer set following two criteria: (a) coming from non-heterozygous contigs (coverage > 60); (b) having multiple copies in all the contig sequence. After the above filtering process, the final set of heterozygous 19-mers in the genome was determined and their frequency distribution is shown in **Fig C**. We also performed a k-mer analysis of the PE reads by sequencing a ‘Dongzao’ mature tree (**Table W in S2 File**).

We then selected contigs and reads which contained the final heterozygous 19-mer set and consider them as heterozygous contigs and heterozygous reads. SOAPdenovo were used to generate links between heterozygous contigs by mapping heterozygous reads back to them and a minimum of three read pairs were required to define an effective link. The links contained orientation and distance information between heterozygous contigs. In a bubble structure, two contigs represented two potential haploids in a diploid genome and one of them would be discarded in SOAPdenovo to extend the continuity of contigs. With the links between heterozygous contigs, we discarded additional contigs, which were considered to be the same haploid as the discarded contigs from bubbles. After solving the bubble structures and the heterozygous regions related to them, we improved the initial contig N50 from 304 bp to 875 bp.

Next, all of the short-insert paired-end and large-insert mate-pair reads were aligned back onto the contig sequences using SOAPdenovo. Contigs with high coverage usually represented the collapse of repetitive sequences with high similarity. These repeat contigs would be masked as they tend to have conflicting connections to other contigs. As implemented in SOAPdenovo, a hierarchical strategy was adopted to generate compatible connections for scaffolding of contigs by adding data from short-insert reads to long-insert reads step by step. Finally, all the short-insert reads were realigned back onto the scaffold sequences to fill gaps between contigs. Specifically, read pairs with mapping positions around the gap regions were collected to perform a local assembly to finish the gap closure.

## 6. Quality assessment of genome assemblies

High quality reads from short insert size PE libraries, which corresponded to 50x coverage, were aligned to the draft assembly of the ‘Junzao’ genome using BWA [8] with the parameters “-o 1 -i 15”. The completeness of the genome assembly was measured by the distribution of the sequencing depth at each position. Publicly available EST data of *Z. jujuba* was downloaded from NCBI (http://www.ncbi.nlm.nih.gov/nucest/?term=Ziziphus + jujuba, downloaded on Dec 2013). In total, 2,901 EST sequences were mapped to the ‘Junzao’ genome using BLAT [9] with an identity cutoff of 90% (**Table C in S2 File**). A total of 33,441 unigenes assembled by the Trinity package using transcriptome data from the fruit samples were also mapped to the genome (**Table C in S2 File**). The CEGMA pipeline [10] was further used to evaluate the gene region assembly of the ‘Junzao’ and ‘Dongzao’ genomes, as well as the assembled apple and pear genomes (**Fig F**). In addition, we used the BUSCO [11] to assess the quality of the two jujube genome assemblies.

## 7. Repeat annotation

Tandem repeats were identified in the ‘Junzao’ genome using Tandem Repeats Finder (TRF) [12]. *De novo* transposable element (TE) prediction was performed using RepeatModeler (http://www.repeatmasker.org/RepeatModeler.html) with default parameters. A custom library (a combination of Repbase and the non-redundant *de novo* identified TEs) were used to screen the ‘Junzao’ genome using RepeatMasker for DNA-level repeat identification. RepeatProteinMask was used to identify repeat sequences at the protein level.

LTR retro-transposons (LTR-RTs) were identified by searching the genomes of ‘Dongzao’ and ‘Junzao’ with LTR_Finder [13]. All identified LTR-RTs were subsequently annotated by BLASTX against the GyDB database [14]. Based on the order of reverse transcriptase (RT) and integrase (INT) in the DNA polymerase (POL) [15], we classified LTR-RTs into two subfamilies: *Copia* and *Gypsy*.

To estimate LTR-RT divergence rates, we use EMBOSS program distmat [16] to calculate the divergence between the 5’- and 3’- LTR sequences. Reverse transcriptases of LTR-RTs in the two jujube genomes were used to construct phylogenetic trees with rice as the outgroup. Multiple protein sequence alignments of reverse transcriptases were performed using MUSCLE [17], and the neighbor-joining (NJ) phylogenetic trees for *Copia* and *Gypsy* were constructed using TreeBeST (http://treesoft.sourceforge.net/treebest.shtml).

## 8. Gene annotation

All predicted *Z. jujuba* ‘Junzao’ genes were functionally annotated by comparing their protein sequences against the SwissProt and TrEMBL [18] databases using BLASTP. Protein domains were identified by comparing the proteins to the InterPro database using InterProScan [19]. Gene Ontology [20] and KEGG metabolic pathways [21] were further assigned to the ‘Junzao’ predicted genes.

# Supplementary Figures


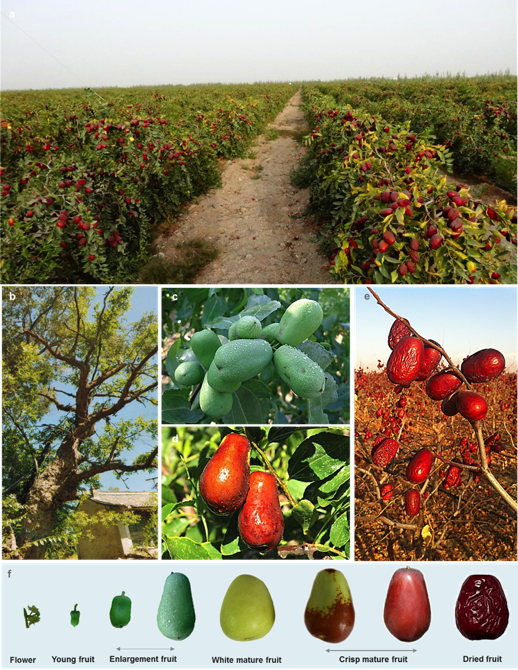


**Fig A. Cultivation and morphological characteristics of the dry cultivar ‘Junzao’.** (a) Cultivation of ‘Junzao’ in arid desert conditions with a wide row planting pattern (3.5m × 1m). (b) A ‘Junzao’ tree of more than 600 years old, in Jiaocheng, Shanxi Province. (c) Developing fruits at the expanding stage; (d) Fruits at the full red mature stage. (e) Naturally dried fruits after fully maturing on the tree. (f) Major development stages of jujube fruit and dried fruit after fully maturing.


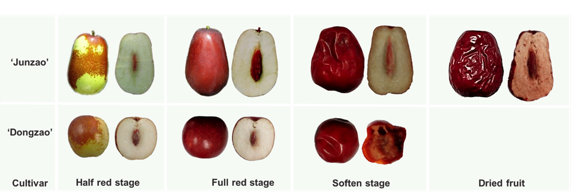


**Fig B. Fruits during ripening and post-harvest storage of a typical dry cultivar (‘Junzao’) and a fresh cultivar (‘Dongzao’).** During the softening stage, fruits of the dry-cultivar ‘Junzao’ shrink while fruits of the fresh cultivar ‘Dongzao’ decay and do not reach the dried stage.


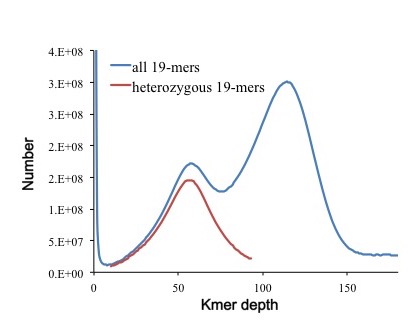
,

**Fig C. Frequency distribution of all 19-mers and heterozygous 19-mers of ‘Junzao’.**


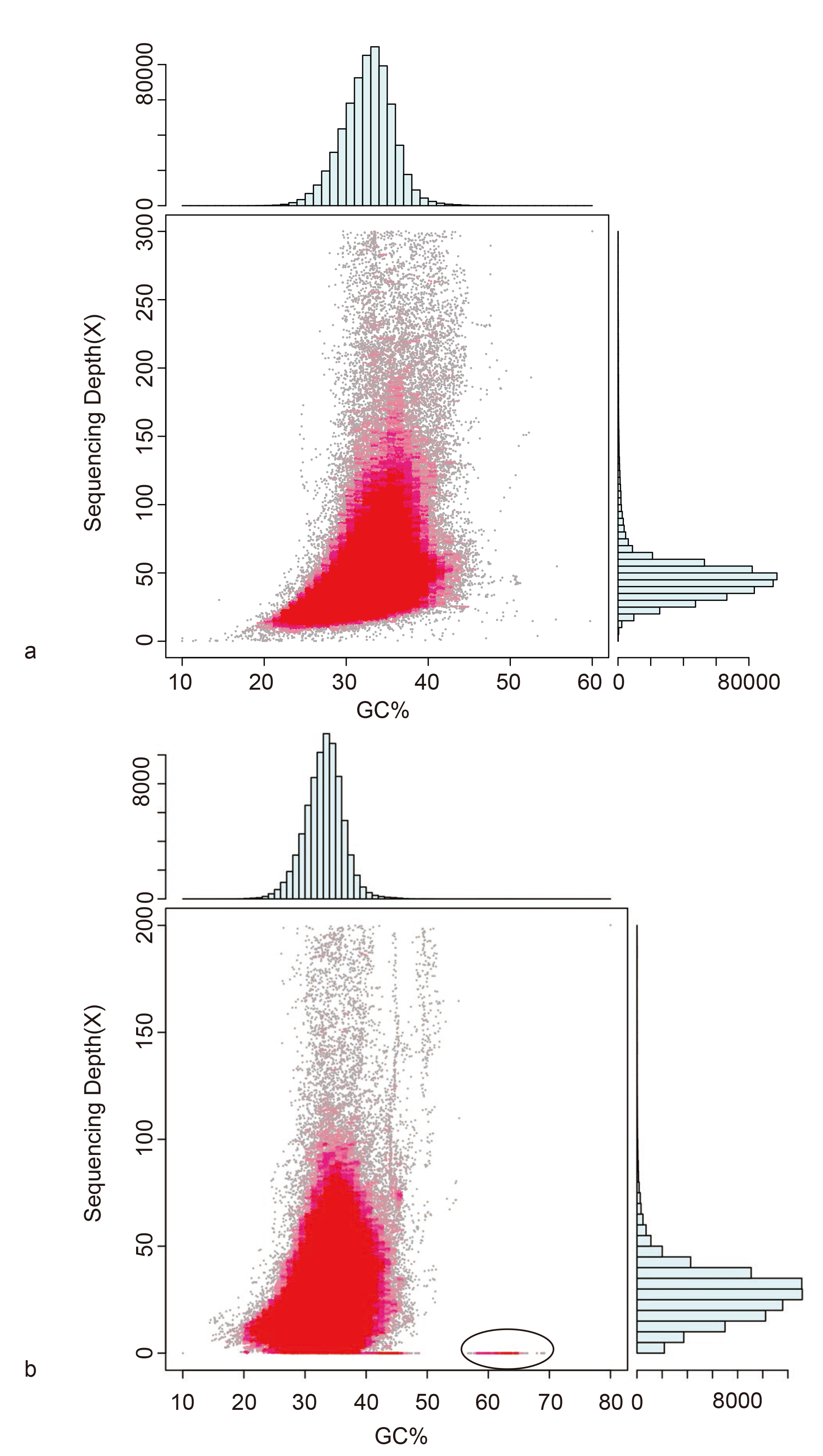


**Fig D. GC content and sequencing depth.** (a) A major island in the scatter graph of the distribution of GC content against sequencing depth indicated no contamination from other species in ‘Junzao’, (b) A small cluster (indicated with a circle) distant from the major island presented in ‘Dongzao’.


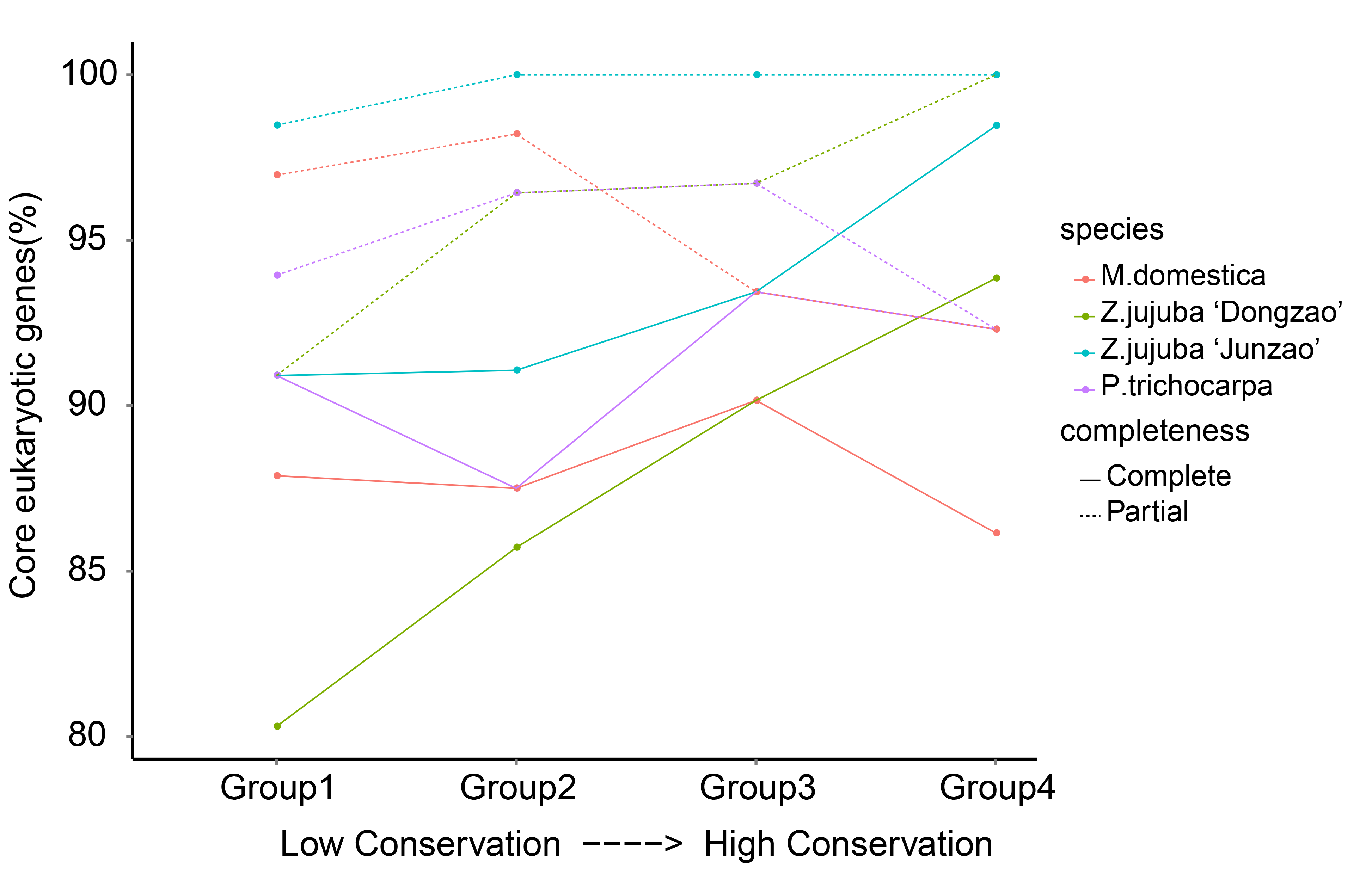


**Fig E. Mapping results of four core eukaryotic gene subsets to the genomes ‘Junzao’, ‘Dongzao’, *M.* x *domestica* and *P. trichocarpa***. The total set of core eukaryotic genes was divided into four groups according to their degree of protein sequence conservation.


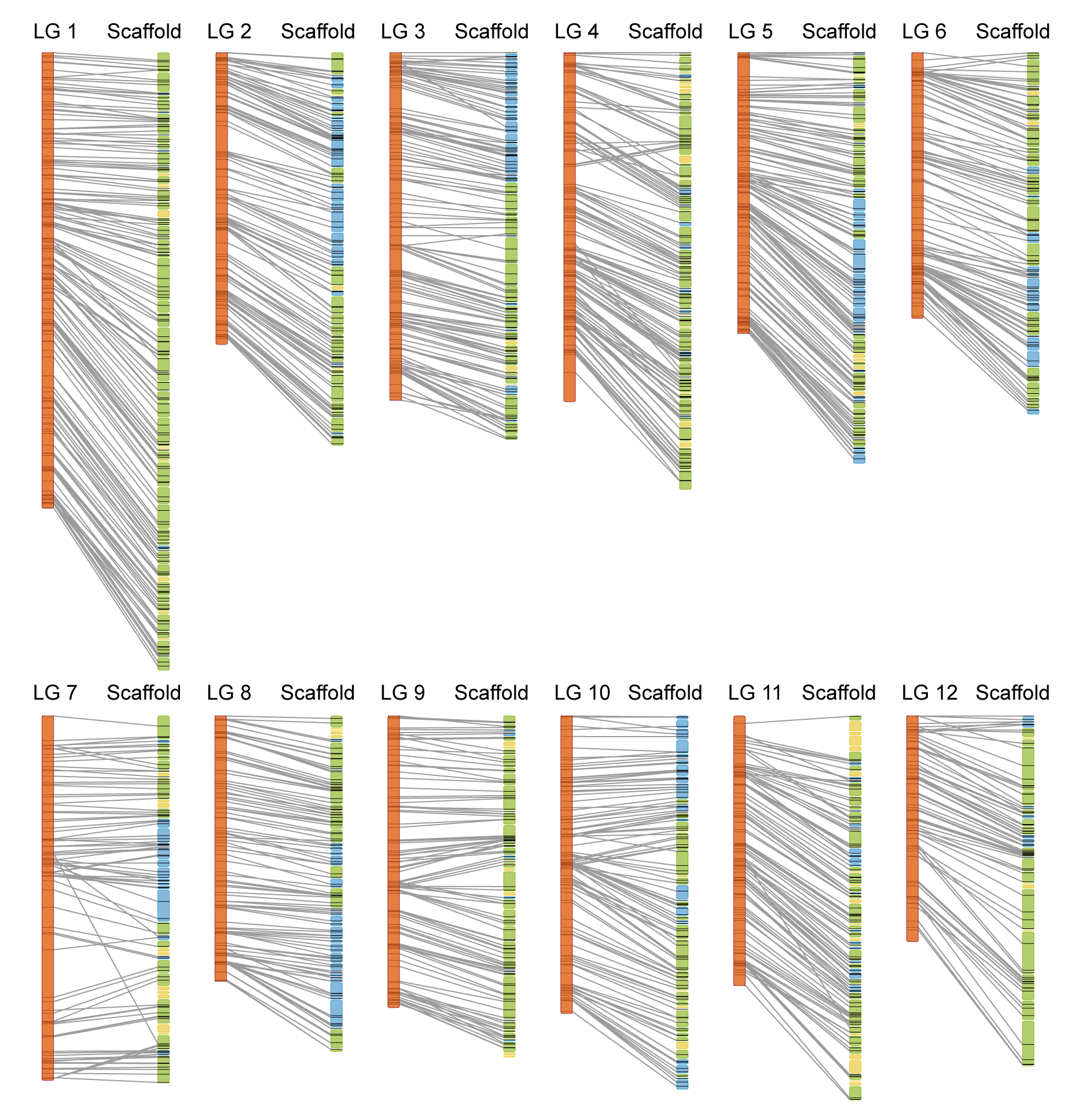


**Fig F. Anchoring the ‘Junzao’ assembled scaffolds to genetic maps.** The ‘Junzao’ assembled scaffolds were anchored to the 12 linkage groups (LG1-LG12, red) using two high-density genetic linkage maps. A total of 208 Mb (green, 59.28% of the assembled genome) were anchored by both maps, 71 Mb (Blue, 20.48%) were anchored only by the genetic map reported by Zhao et al. [22], and 13 Mb (yellow, 3.86%) were anchored only by the genetic map constructed in this study.


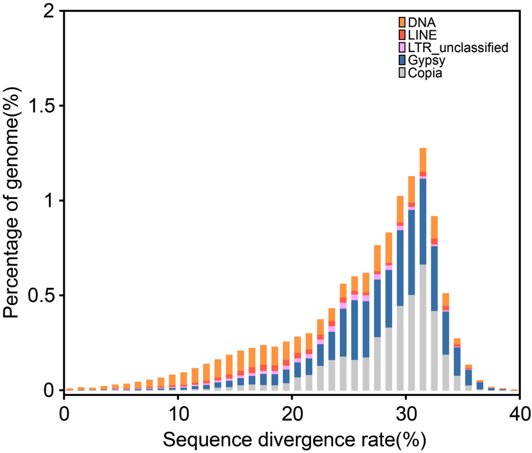


**Fig G. Divergence rate of transposable elements in the *Z. jujuba* ‘Junzao’ genome.**

**
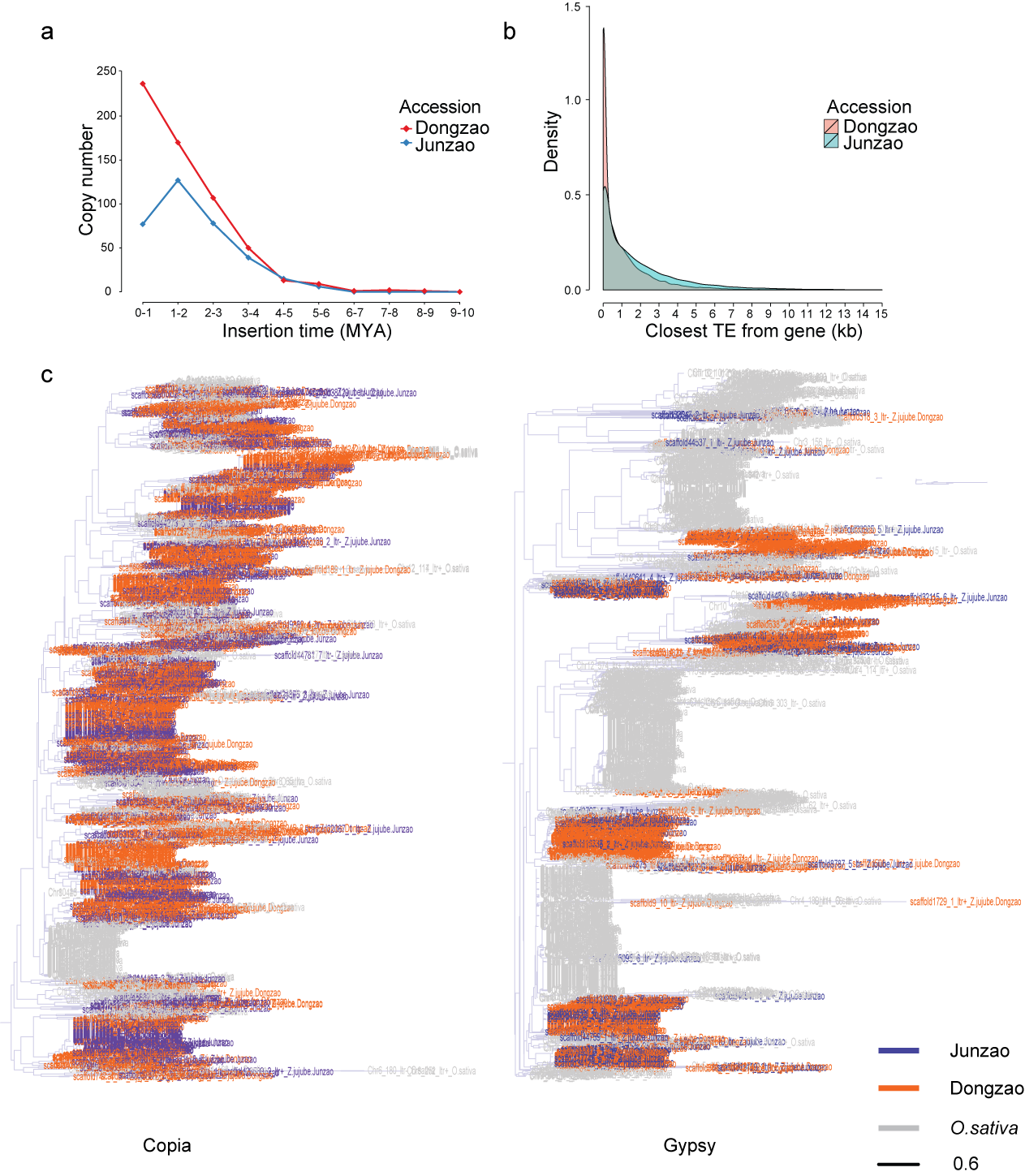
Fig H. Comparison of transposable elements between the two genome sequences of *Ziziphus jujuba*.** (a) Insertion time of long terminal repeat (LTR) retrotransposons in the ‘Dongzao’ and ‘Junzao’ genomes. The insertion time was estimated using the formula: T (time) = K/(2*r), where K represents the average number of substitutions per aligned site and r represents the average substitution rate, which was assigned as 1.3e-8 substitutions per synonymous site per year. (b) Distance from individual TEs to their closest genes. (c) Phylogeny of Ty1/copia-like and Ty3/gypsy-like LTR retrotransposons.


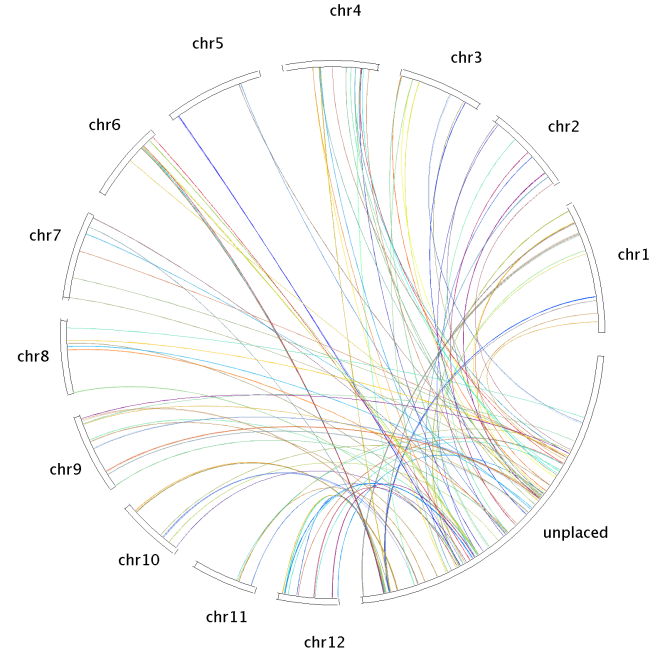

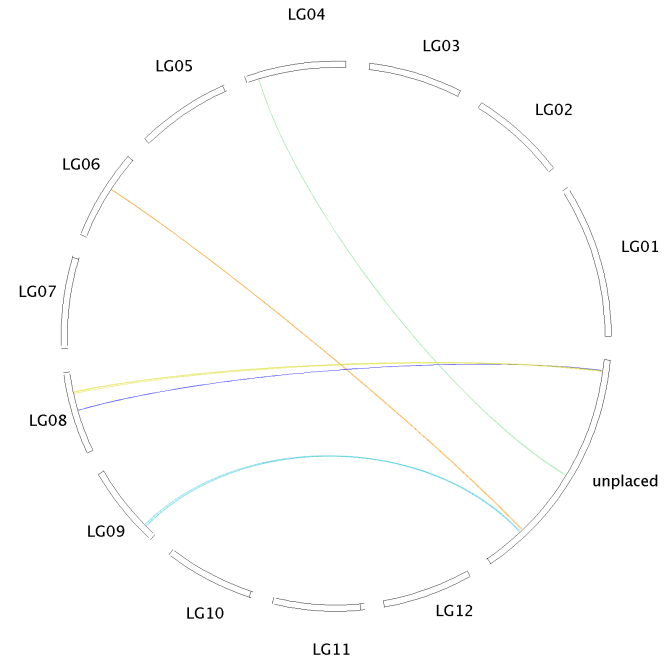


**Fig I.** **Orthologous gene blocks between anchored scaffolds and unanchored scaffolds in ‘Dongzao’ (left) and ‘Junzao’ (right)**.


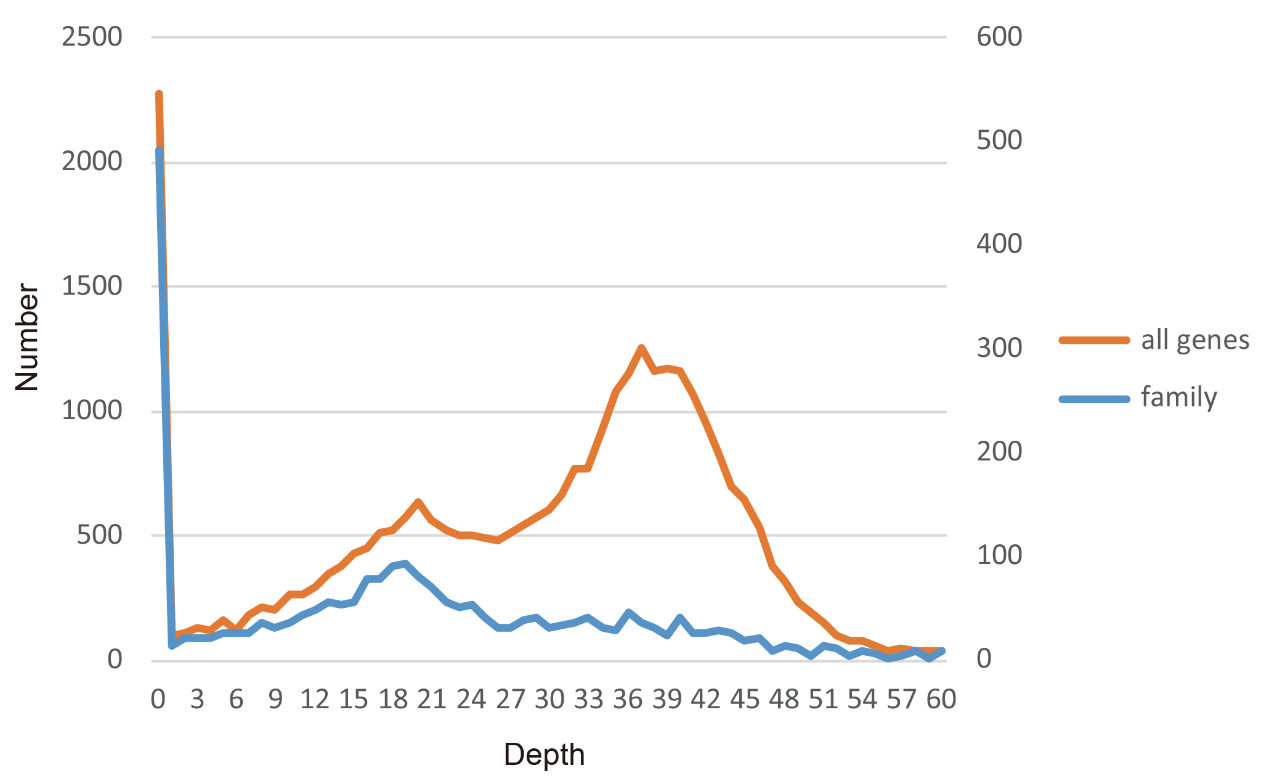


**Fig J. Average sequencing depth distribution of ‘Dongzao’ genes**. All the cleaned reads generated by sequencing genomic DNA derived from a mature ‘Dongzao’ tree were mapped to the assembled ‘Dongzao’ genome and the average sequencing depth of genes was plotted (orange). For most of genes, the sequencing depth was 36×. However, we observed a secondary peak in the distribution at half of the average sequencing depth (18×). The average sequencing depth of the 2,615 genes from 1,126 families containing fewer gene members in ‘Junzao’ than ‘Dongzao’ was ~18× (blue).


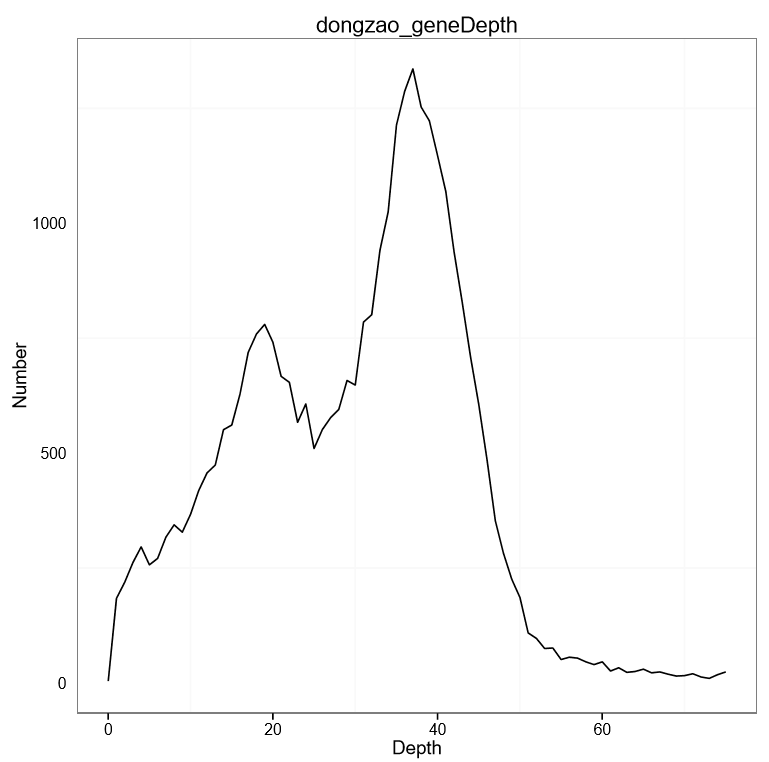

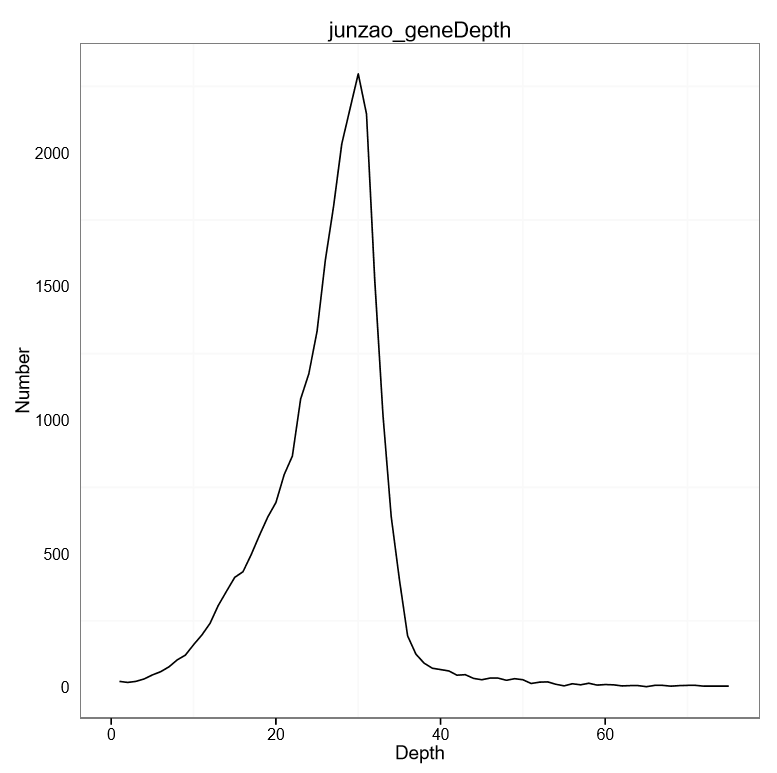


**Fig K. Read coverage distributions of coding regions in ‘Junzao’ and the previous reported ‘Dongzao’.** ‘Dongzao’ reads were generated by sequencing genomic DNA derived from a mature ‘Dongzao’ tree and mapped to the previous reported ‘Dongzao’ gene models as described in S10.


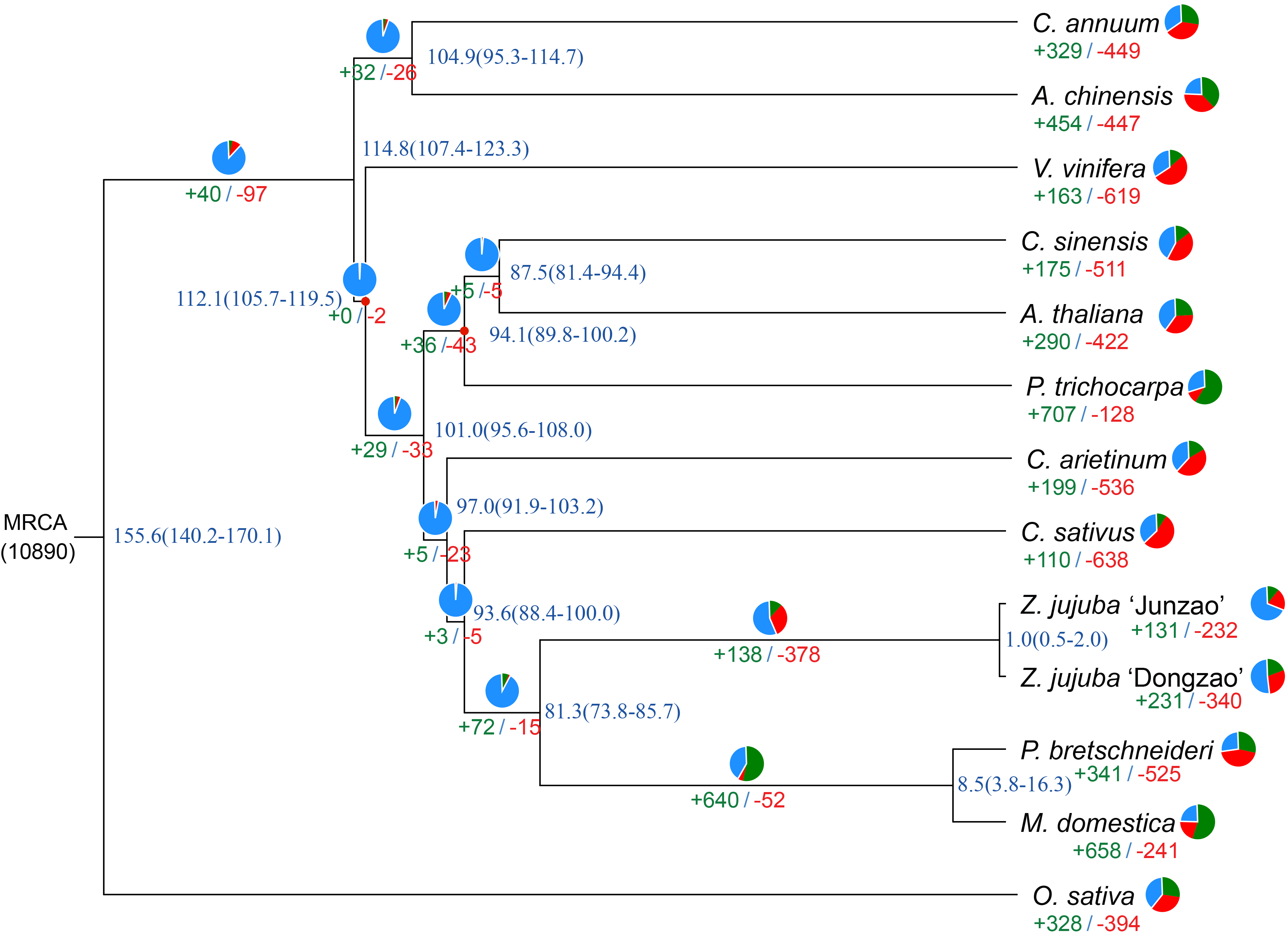


**Fig L. Phylogenetic tree and gene family expansion and contraction.** The phylogenetic tree was constructed from a concatenated alignment of 205 single-copy gene families from 12 eudicots and *O. sativa*. Gene family expansions are indicated in green, and contractions are indicated in red; the corresponding proportions among total changes are shown using the same colors in the pie charts.

**
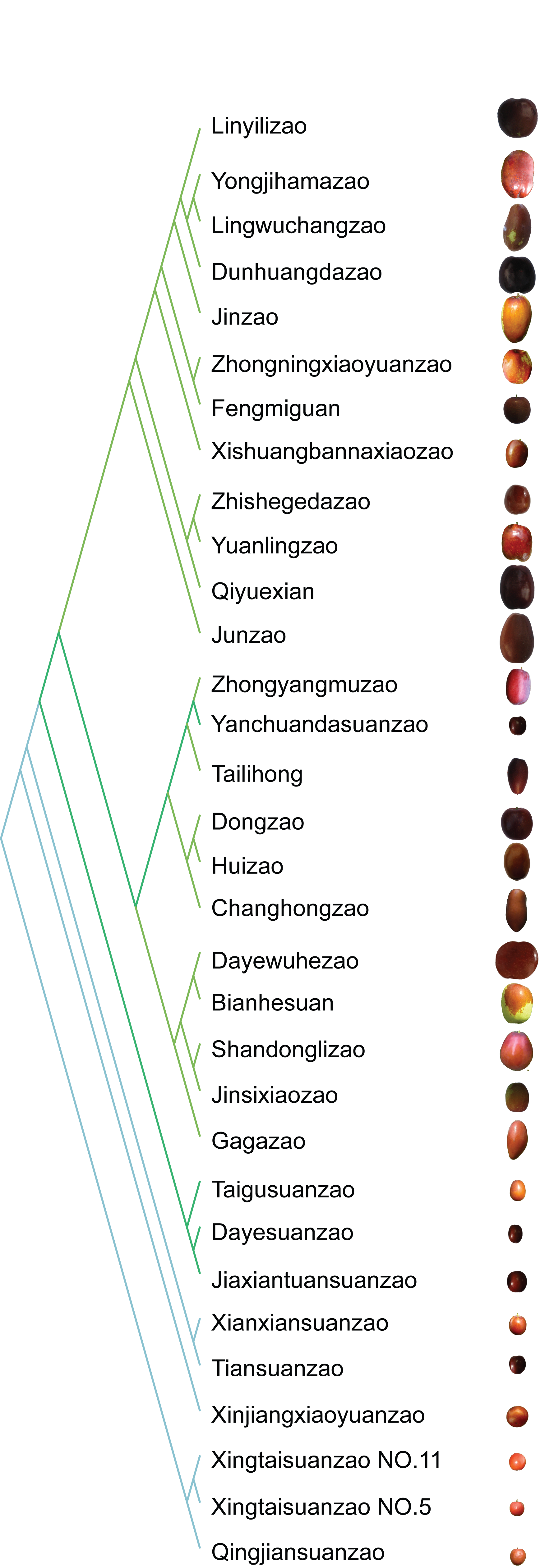
Fig M. Fruits of the jujube accessions used the in phylogenetic analysis.**

**
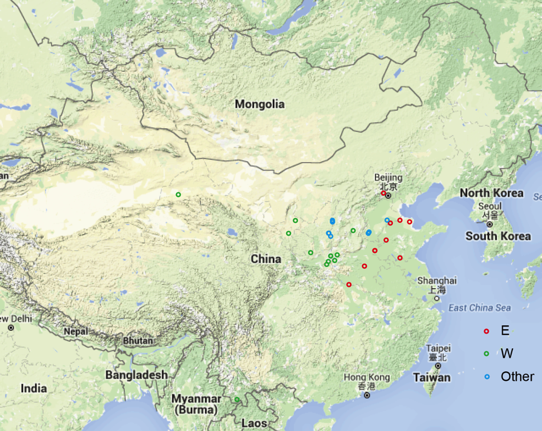
**

**Fig N. Geographical location of the jujube cultivars and wild jujube accessions used in the resequencing analyses.** Cultivated jujubes sampled from East China are marked by red circles (E), those from West China by green circles (W), and the wild accessions by blue circles (Other).

**
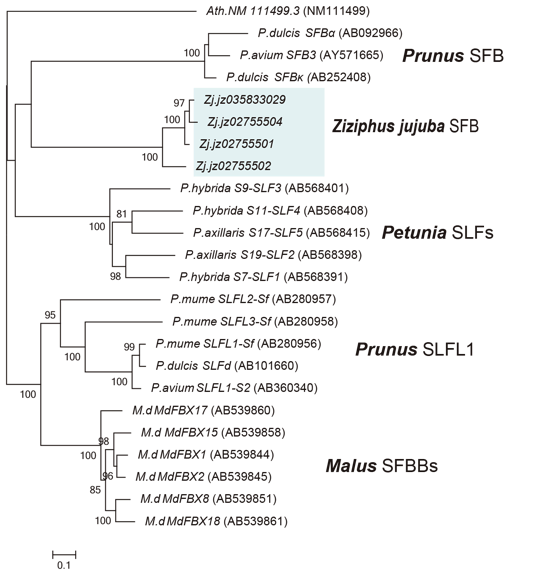
**

**Fig O. Phylogenetic tree of predicted SFB genes in Z. jujuba and SFB, SLF and SLF-like genes identified in *Malus* x *domestica*, *Prunus persica*, *Prunus mume*, *Fragaria vesca*, and *Fragaria nipponica*.** The tree was rooted with *A. thaliana* F-box/kelch-repeat gene (*NM111499*). Phylogenetic tree was constructed using RAxML with the Generalised Time-Reversible (GTR) model of sequence evolution.


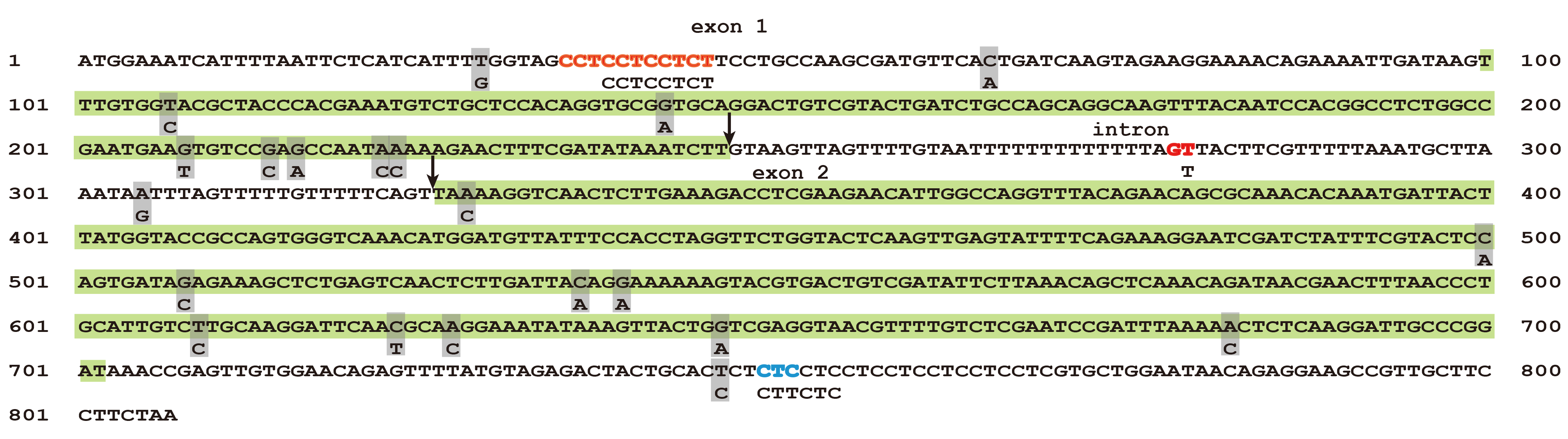


**Fig P. SNPs and indels identified in the candidate S-RNase gene (*Zj.jz035833030*) based on resequencing results.** Bases with green background indicate the Ribonuclease domain of T2-RNase.

# References

1. Song FH, Yishake H, Shi YJ, Zhang P, Luo QH. Correlation analysis between soil nutrient and fruit quality of *Ziziphus jujuba* cv. Junzao in Tarim Basin of Xinjiang. J Fruit Sci. 2010;27:626-630.

2. Li D, Niu X, Tian J. The illustrated germplasm resources of Chinese jujube. Beijing: China Agricultural Press; 2013.

3. Ji SS. Studies on the functional sugar from Chinese jujube. Agricultural University of Hebei, 2012.

4. Chen JP, Li ZG, Maitinuer M, Zhang WL, Zhan JYX, et al. Chemical and biological assessment of *Ziziphus jujuba* fruits from China: different geographical sources and developmental stages. J Agr Food Chem. 2013;61:7315-7324.

5. Kao TH, Chen B. Functional components in *Zizyphus* with emphasis on polysaccharides. In polysaccharides bioactivity and biotechnology (Ramawat KG, Merillon JM eds.), vol. 1. pp. 795-821: Springer International Publishing; 2015:795-821.

6. Huang H, Tong, Y, Zhang QJ, Gao LZ. Genome size variation among and within *Camellia* species by using flow cytometric analysis. PLoS ONE. 2013;8: e64981.

7. Li R, Zhu H, Ruan J, Qian W, Fang X, et al. De novo assembly of human genomes with massively parallel short read sequencing. Genome Res. 2010;20:265-272.

8. Li H, Durbin R. Fast and accurate short read alignment with Burrows-Wheeler transform. Bioinformatics. 2009;25:1754-1760.

9. Kent WJ. BLAT-the BLAST-like alignment tool. Genome Res. 2002;12:656-664.

10. Parra G, Bradnam K, Korf I. CEGMA: a pipeline to accurately annotate core genes in eukaryotic genomes. Bioinformatics. 2007;23:1061-1067.

11. Simao FA, Waterhouse RM, Ioannidis P, Kriventseva EV, Zdobnov EM. BUSCO: assessing genome assembly and annotation completeness with single-copy orthologs. Bioinformatics. 2015;31:3210-3212.

12. Chen N. Using RepeatMasker to identify repetitive elements in genomic sequences. Curr Protoc Bioinformatics. 2004;Chapter 4:Unit 4 10.

13. Xu Z, Wang H. LTR_FINDER: an efficient tool for the prediction of full-length LTR retrotransposons. Nucleic Acids Res. 2007;35:W265-268.

14. Llorens C, Futami R, Covelli L, Dominguez-Escriba L, Viu JM, et al. The gypsy database (GyDB) of mobile genetic elements: release 2.0. Nucleic Acids Res. 2011;39:D70-74.

15. Wicker T, Sabot F, Hua-Van A, Bennetzen JL, Capy P, et al. A unified classification system for eukaryotic transposable elements. Nat Rev Genet. 2007;8:973-982.

16. Rice P, Longden I, Bleasby A. EMBOSS: the European molecular biology open software suite. Trends Genet. 2000;16:276-277.

17. Edgar RC. MUSCLE: multiple sequence alignment with high accuracy and high throughput. Nucleic Acids Res. 2004;32:1792-1797.

18. Bairoch A, Apweiler R. The SWISS-PROT protein sequence database and its supplement TrEMBL in 2000. Nucleic Acids Res. 2000;28:45-48.

19. Mulder N, Apweiler R. InterPro and InterProScan: tools for protein sequence classification and comparison. Methods Mol Biol. 2007;396:59-70.

20. Ashburner M, Ball CA, Blake JA, Botstein D, Butler H, et al. Gene ontology: tool for the unification of biology. The Gene Ontology Consortium. Nat Genet. 2000;25:25-29.

21. Kanehisa M, Goto S. KEGG: kyoto encyclopedia of genes and genomes. Nucleic Acids Res. 2000;28:27-30.

22. Zhao J, Jian J, Liu G, Wang J, Lin M, Ming Y, et al. Rapid SNP discovery and a RAD-based high-density linkage map in jujube (Ziziphus Mill.). PLoS ONE 2014;9:e109850.
